# Galacto-oligosaccharides alleviate lung inflammation by inhibiting NLRP3 inflammasome activation *in vivo* and *in vitro*

**DOI:** 10.1016/j.jare.2021.10.013

**Published:** 2021-11-01

**Authors:** Yang Cai, Myrthe S. Gilbert, Walter J.J. Gerrits, Gert Folkerts, Saskia Braber

**Affiliations:** a Division of Pharmacology, Utrecht Institute for Pharmaceutical Sciences, Faculty of Science, Utrecht University, Utrecht, the Netherlands; b Animal Nutrition Group, Wageningen University, Wageningen, the Netherlands

**Keywords:** Respiratory infections, *Mannheimia haemolytica*, IL-1β, Non-digestible oligosaccharides, Primary bronchial epithelial cells, Reactive oxygen species, ATP, adenosine triphosphate, BALF, broncho-alveolar lavage fluid, COS, chitosan-oligosaccharides, DP, degree of polymerization, GOS, galacto-oligosaccharides, HMOs, human milk oligosaccharides, IL, interleukin, LDH, lactate dehydrogenase, LPS, lipopolysaccharides, MR, milk replacer, MDA, malondialdehyde, MTT, thiazolyl blue tetrazolium bromide, MAPK, mitogen-activated protein kinase, NLRP3, NLR family pyrin domain containing 3, NDOs, Non-digestible oligosaccharides, NF-κB, nuclear factor kappa B, NAC, acetylcysteine, PBECs, primary bronchial epithelial cells, ROS, reactive oxygen species, RCrL, right cranial lobe, TLR4, Toll-like receptor 4

## Abstract

**Introduction:**

The lack of effective anti-inflammatory therapies for pneumonia represents a challenge for identifying new alternatives. Non-digestible galacto-oligosaccharides (GOS) are attractive candidates due to their anti-inflammatory and immunomodulatory effects both locally and systemically.

**Objectives:**

The anti-inflammatory properties of GOS were investigated in calves with lung infections and in calf primary bronchial epithelial cells (PBECs) and human lung epithelial cells (A549). To delineate the mechanism, the potential capacity of GOS to inhibit the NLR family pyrin domain containing 3 (NLRP3) inflammasome has been investigated.

**Methods:**

GOS were administrated orally to calves with naturally occurring lung infections during early life or used as pretreatments in cell cultures exposed to *M. haemolytica*, lipopolysaccharides (LPS), leukotoxin or ATP. The cell composition, cytokine/chemokine concentrations, and *M. haemolytica*-LPS lgG levels in broncho-alveolar lavage fluid (BALF) and blood were investigated, while the *M. haemolytica* positivity in BALF and bronchial mucosa was detected *in vivo*. Key markers of NLRP3 inflammasome activation were measured *in vivo* and *in vitro*.

**Results:**

GOS reduced *M. haemolytica* positivity and *M. haemolytica*-LPS lgG levels in calves with lung infections. Regulation of immune function and suppression of inflammatory response by GOS is related to the inhibition of NLRP3 inflammasome as observed in bronchial mucosal tissue of infected calves. The *M. haemolytica*-induced IL-1β production in PBECs was lowered by GOS, which was associated with NLRP3 inflammasome inhibition caused by the decreased reactive oxygen species and ATP production. GOS inhibited leukotoxin-induced ATP production in PBECs. The LPS- and ATP-induced NLRP3 inflammasome activation in PBECs and A549 cells was suppressed by GOS.

**Conclusion:**

GOS exert anti-inflammatory properties by inhibiting the NLRP3 inflammasome activation *in vitro* and *in vivo*, suggesting a potential role for GOS in the prevention of lung infections.

## Highlights

• GOS suppress both local and systemic inflammation in lung infections.
• GOS reduce the *M. haemolytica* positivity in calves with lung infections.
• GOS inhibit NLRP3 inflammasome activation in vivo and in vitro.
• GOS decrease ATP production in PBECs induced by *M. haemolytica*.
• Direct anti-oxidative effects of GOS on lung cells are involved.

## Graphical abstract

**Figure:**
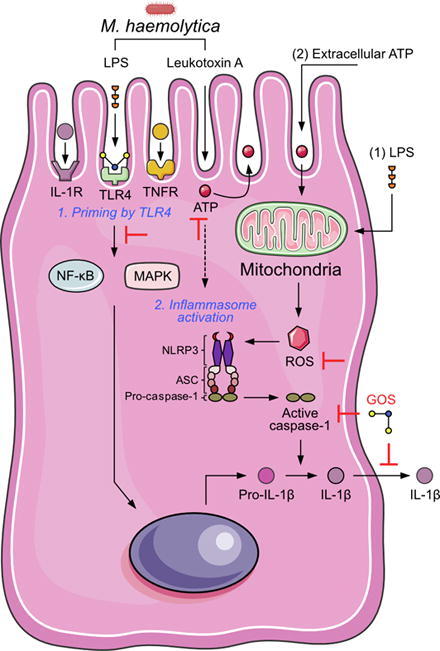

## Introduction

Lung infection is the single biggest cause of pediatric death worldwide [1] and one of the most common causes of morbidity and mortality in calves [2]. The calf is considered to be a valuable animal model for studying lung infections, due to the extremely high natural prevalence, easy-to-observe respiratory symptoms, repetitive sampling *in vivo*, and the continuous production of proinflammatory mediators in the airways [3], [4]. *Mannheimia haemolytica* is one of the principal Gram-negative bacteria in calves that causes lung infections characterized by a decline in innate immune function, dysfunction of airway epithelium and a large influx of inflammatory mediators (e.g., IL-1β, TNF-α) into the airways [2], [5]. As immune sentinels, lung epithelial cells detect inhaled pathogens through ample pattern-recognition receptors, such as Toll-like receptors (TLRs) and NOD-like receptors (NLRs) [6]. These receptors can recognize virulence factors of pathogens, for example, *M. haemolytica* can release lipopolysaccharides (LPS) to activate TLR4 [3]. Despite extensive research, antibiotics remain the mainstay for the treatment of lung infections.

Recently, it was found that NLR family pyrin domain containing 3 (NLRP3) inflammasome is strongly involved in lung inflammation and infection in humans and rodents [1]. The NLRP3 inflammasome is a unique inflammasome, whose activation is a two-step process induced by various microbial molecules (e.g., LPS) or danger signals (e.g., ROS, ATP) [1]. Firstly, a priming event induced by “TLR/NF-κB” signaling increases the expression of NLRP3 and pro-IL-1β. The second signal triggers inflammasome multimerization and IL-1β maturation. NLRP3 inflammasome can be activated by multiple respiratory pathogens, including *S. aureus* [7], *S. pneumoniae* [8] and *K. pneumoniae* [9], which indicates the NLRP3 inflammasome could be a potential therapeutic and preventive target for lung infections [1].

Non-digestible oligosaccharides (NDOs) are a group of low molecular weight carbohydrates. Among them, galacto-oligosaccharides (GOS) are well known NDOs and are composed of a galactose chain (DP 1–5) attached to a single glucose molecule and produced by the conversion of lactose by β-galactosidase [10]. NDOs have the potential to prevent respiratory diseases due to their prebiotic, anti-inflammatory and immunomodulatory effects [11], [12]. There are some indications that NDOs could probably be effective against respiratory infections [13], [14], [15], [16]. Oral acidic oligosaccharides derived from pectin increased bacterial clearance in mice with a *P. aeruginosa*-induced lung infection [13]. NDO mixtures containing GOS and fructo-oligosaccharides prevented, particularly, respiratory infections during the first 6 months of age [14] and reduced the frequency of respiratory infections, and antibiotic prescriptions in the first two years of life [15]. In addition, GOS supplementation decreased the duration and symptoms of cold or flu among university students [16]. More mechanism-related research is required to investigate the possibility of using GOS as a strategy to prevent respiratory infections.

Here, we investigated whether GOS would alleviate airway inflammation in calves with lung infections and tried to unravel the mechanism by using *in vitro* systems with calf primary bronchial epithelial cells (PBECs) and human lung epithelial cells stimulated with *M. haemolytica*/LPS/ATP*.* For the first time, the importance of NLRP3 inflammasome activation in a bovine lung infection has been demonstrated, whereas GOS mitigated the infection-induced inflammatory response, which might be explained by the inhibition of the NLRP3 inflammasome activation. GOS supplementation with or without the combination of standard drugs might be a promising future strategy to combat respiratory infections.

## Results

### Remission of lung infections and inhibition of inflammation by GOS

**Fig. 1:**
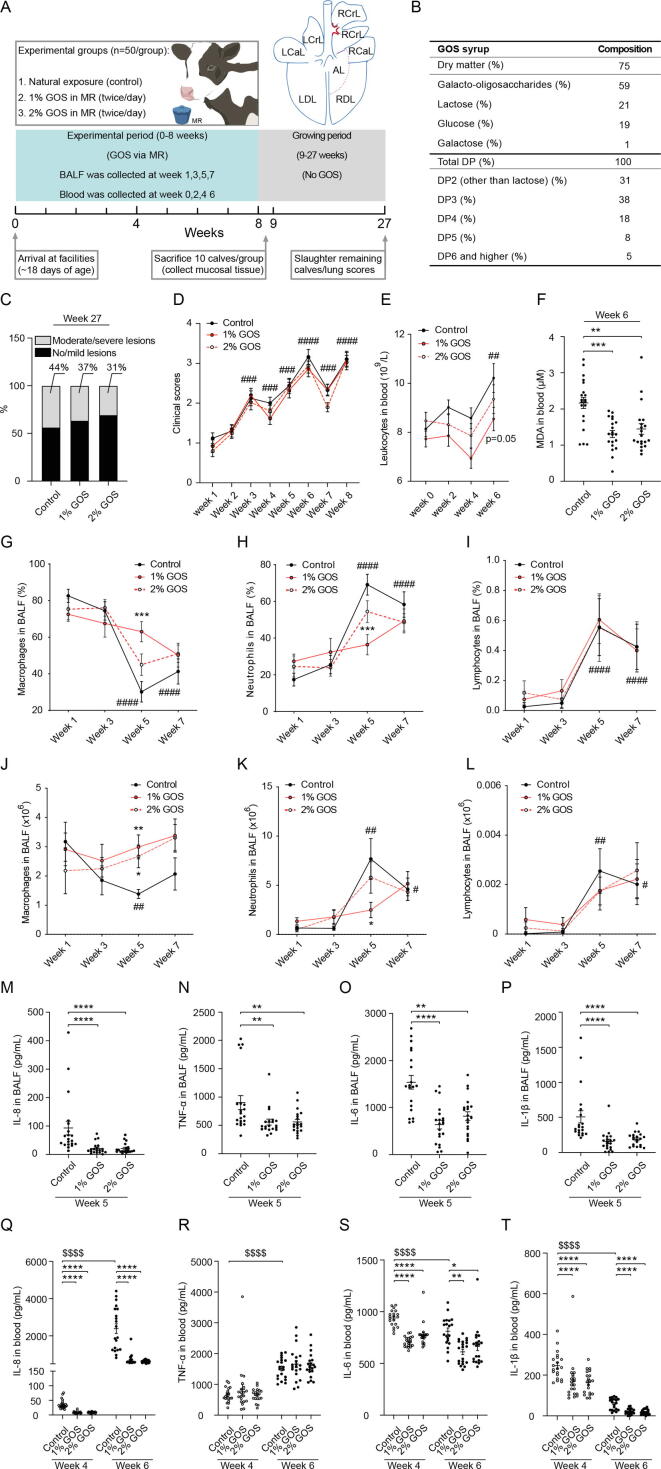
**Effect of GOS on lung and clinical scores as well as on cell composition and cytokine/chemokine measurements in BALF and blood.** (**A**) Timeline and design of the experiment. Calves were naturally exposed to respiratory pathogens in the environment and treated twice per day with or without GOS orally for 8 weeks during early life followed by 19 weeks (week 9–27) without GOS administration. BALF and blood were collected at week 1, 3, 5, 7 and week 0, 2, 4, 6, respectively. Bronchial mucosal tissue (red area around the right cranial lobe) was collected at week 8. Lung scores were assessed at week 27. (**B**) GOS syrup composition. (**C**) Proportion of different lung lesions in calves was calculated based on the lung scores at week 27 (n = 107, 34–37 calves/group). (**D**) Clinical scores were evaluated over time (n = 150, 50 calves/group). (**E**) Concentrations of leukocytes in blood were measured at week 0, 2, 4, and 6 (n = 60, 20 calves/group). (**F**) MDA release in blood was assessed at week 6 (n = 60, 20 calves/group). (**G**-**L**) Percentages and numbers of macrophages, neutrophils, and lymphocytes in BALF were measured at week 1, 3, 5 and 7 (n = 60, 20 calves/group). (**M**-**P**) Concentrations of IL-8, TNF-α, IL-6, and IL-1β in BALF were measured by ELISA at week 5 (n = 60, 20 calves/group). (**Q**-**T**) Levels of IL-8, TNF-α, IL-6, and IL-1β in blood were determined by ELISA at week 4 and 6 (n = 60, 20 calves/group). *P* = 0.05, *P < 0.05, **P < 0.01, ***P < 0.001, ****P < 0.0001 (GOS treatments vs control group); #*P* < 0.05, ##*P* < 0.01, ###*P* < 0.001, ####*P* < 0.0001 (control week 3–8 vs week 0 or 1); $$$$*P* < 0.0001 (control week 6 vs week 4). Data are presented as means ± SEM. Each dot represents one calf. AL = accessory lobe; BALF = broncho-alveolar lavage fluid; DP = degree of polymerization; GOS = galacto-oligosaccharides; IL = interleukin; LCrL = left cranial lobe; LCaL = left cardiac lobe; LDL = left diaphragmatic lobe; MR = milk replacer; MDA = malondialdehyde; RCrL = right cranial lobe; RCaL = right cardiac lobe; RDL = right diaphragmatic lobe; TNF = tumor necrosis factor.

All calves were naturally exposed to respiratory pathogens in the environment. GOS were orally (twice/day) administrated to calves from experimental week 1 till 8 to manipulate early-life conditions, thereafter the animals were followed from week 9 till 27 without GOS administration (Fig. 1A and B). At week 27, all calves received lung scores after slaughter. Oral 2% GOS tended to show lower proportion of moderate/severe lung lesions (31%, p = 0.09) compared with the control group (44%, Fig. 1C). In addition, GOS showed no significant effects on clinical scores, a scoring system for rectal temperature, coughing, nasal discharge and behavior, during the experimental period (Fig. 1D).

To study the extent of lung infection in calves upon GOS administration, the blood and broncho-alveolar lavage fluid (BALF) was examined at week 0, 2, 4, 6 and week 1, 3, 5, 7, respectively (Fig. 1A). Increased leukocytes in the blood and total cells (mainly pulmonary leukocytes) in BALF of control calves were observed at week 6 and 5, respectively (Fig. 1E and supplementary Figure S1A). Decreased percentage and number of macrophages and increased percentage and number of neutrophils and lymphocytes were observed from week 5 in control calves (Fig. 1G-L). Furthermore, the clinical scores increased significantly over time with the peak at week 6 (Fig. 1D). These findings indicate that lung infections are present in these calves from week 5.

Control calves displayed a significant increase in blood leukocyte concentrations, which increased by 26% at week 6 compared with week 0, while 1% GOS tended to (p = 0.05) decrease the concentrations of leukocytes at week 6 (Fig. 1E). Furthermore, GOS revealed a significant reduction in malondialdehyde (MDA) levels (a biomarker for oxidative stress) in blood compared with control calves at week 6 (Fig. 1F).

Dietary GOS significantly increased the percentage and number of macrophages and decreased the percentage and number of neutrophils in BALF at week 5 (Fig. 1G, H, J, and K), compared with the control animals. Interestingly, GOS have no effects on the lymphocytes (Fig. 1I and L). In addition, none of the GOS treatments did affect the total cell numbers in BALF (supplementary Figure S1A). Overall, GOS at 1% restored the imbalance of the ratio of macrophages to neutrophils in BALF caused by lung infections at week 5.

The inflammatory response in the lungs was investigated by measuring proinflammatory cytokines/chemokines in BALF. GOS significantly reduced the concentrations of IL-8, TNF-α, IL-6, and IL-1β in BALF at week 5 (Fig. 1M-P). The same cytokines/chemokines were measured in the blood to investigate the effect of GOS on systemic inflammation caused by lung infections at week 4 and 6. In control group, the IL-8 and TNF-α levels in blood were significantly increased at week 6 compared with week 4, while IL-6 and IL-1β concentrations were slightly reduced. GOS significantly reduced the IL-8, IL-6 and IL-1β levels in the blood at both week 4 and 6 (Fig. 1Q-T).

### Reduction of *M. haemolytica*-LPS lgG levels and *M. haemolytica* positivity in the lungs by GOS

**Fig. 2:**
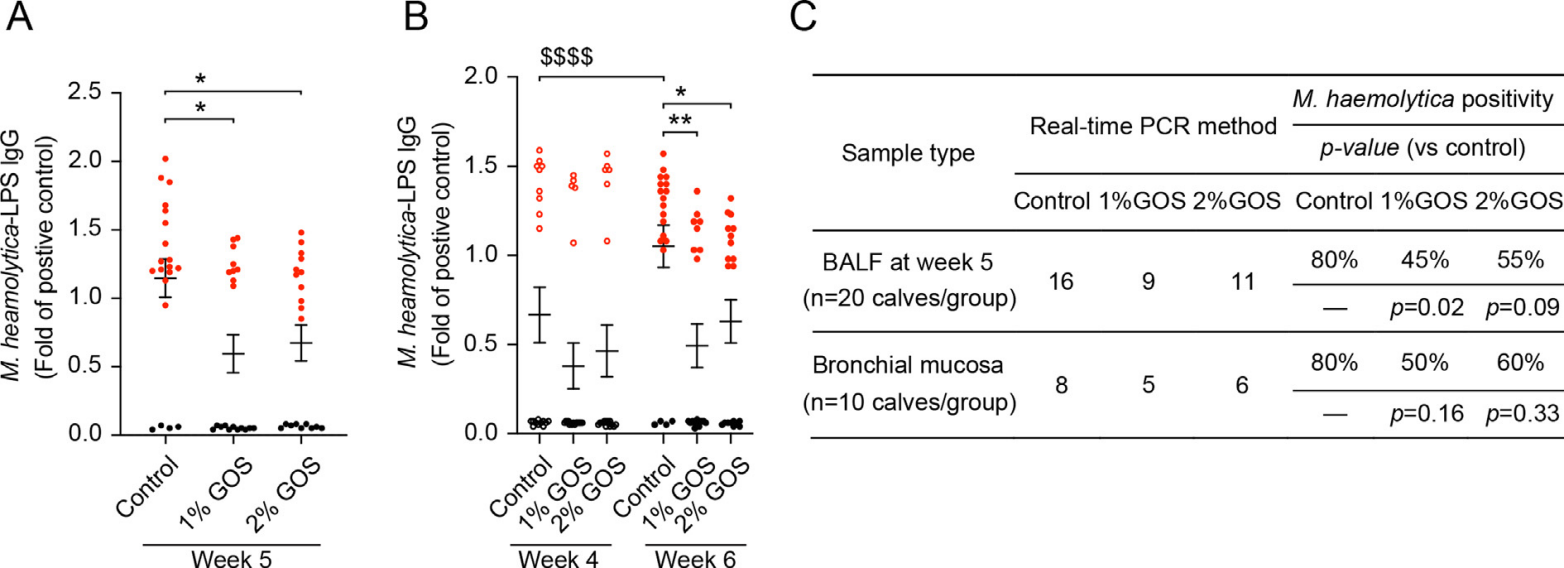
**Reduction of *M. haemolytica*-LPS lgG levels and *M. haemolytica* positivity in the lungs by GOS.** (**A**-**B**) *M. haemolytica*-LPS lgG levels were detected in BALF at week 5 (**A**) and in blood at week 4 and 6 (**B**) by ELISA. Compared to the positive control, fold changes were calculated (n = 60, 20 calves/group). Black dots represent *M. haemolytica*-LPS lgG negative calves. Red dots represent *M. haemolytica*-LPS lgG positive calves. (**C**) Number and percentage of *M. haemolytica* positive calves according to the presence in BALF or bronchial mucosa by real-time PCR method. *P < 0.05, **P < 0.01 (GOS treatments vs control group); $$$$*P* < 0.0001 (control week 6 vs week 4). Data are presented as means ± SEM. BALF = broncho-alveolar lavage fluid; GOS = galacto-oligosaccharides; LPS = lipopolysaccharides.

*M. haemolytica* is one of the main pathogens that contribute to the development of bovine lung infections. It releases LPS to produce proinflammatory cytokines/chemokines and promote the lung lesions through the stimulation of epithelial cells and leukocytes [5]. Here, the *M. haemolytica*-LPS lgG was detected in BALF and blood. Compared to week 1 (0%), the number of calves positive for *M. haemolytica*-LPS lgG within the control group increased over time and reached 80% at week 5 (supplementary Table S1). The effect of GOS on *M. haemolytica*-LPS lgG levels was investigated in BALF at week 5 and in blood at week 4 and 6 (the same timepoint for the measurements of cytokines/chemokines). Interestingly, GOS reduced the *M. haemolytica*-LPS lgG levels in BALF and blood at week 5 and 6, respectively (Fig. 2A-B).

The detection of *M. haemolytica*-LPS lgG is an indirect method for identifying *M. haemolytica* in BALF. Hence, the positivity for *M. haemolytica* was detected by real-time PCR in BALF of all calves at week 5. Fig. 2C showed that 80% (16/20) of control calves were positive for *M. haemolytica*. In line with the data from *M. haemolytica*-LPS lgG detection in BALF (supplementary Table S1), it indicated that *M. haemolytica* might be involved in the naturally occurring lung infections of calves from week 5. Interestingly, administration of 1% and 2% GOS showed a reduction in the number of calves positive for *M. haemolytica* (45% and 55% of the calves were positive for *M. haemolytica* at week 5, respectively) (Fig. 2C).

Infected lesions are most often observed in the right cranial lobe (RCrL) of bovine lungs in previous research [17] and current study as depicted in supplementary Figure S1B. The bronchial mucosal tissue nearby the RCrL was collected (Fig. 1A) and the presence of *M. haemolytica* was identified in these tissues as well. 80% (8/10) of the calves showed that *M. haemolytica* was present in the bronchial mucosa. Although it is not statistically significant, 1% and 2% GOS reduced the number of calves positive for *M. haemolytica* (50% and 60% of the calves were positive for *M. haemolytica* in the bronchial mucosa at week 8, respectively) (Fig. 2C).

### Inhibition of the activation of NLPR3 inflammasome by GOS *in vivo*

**Fig. 3:**
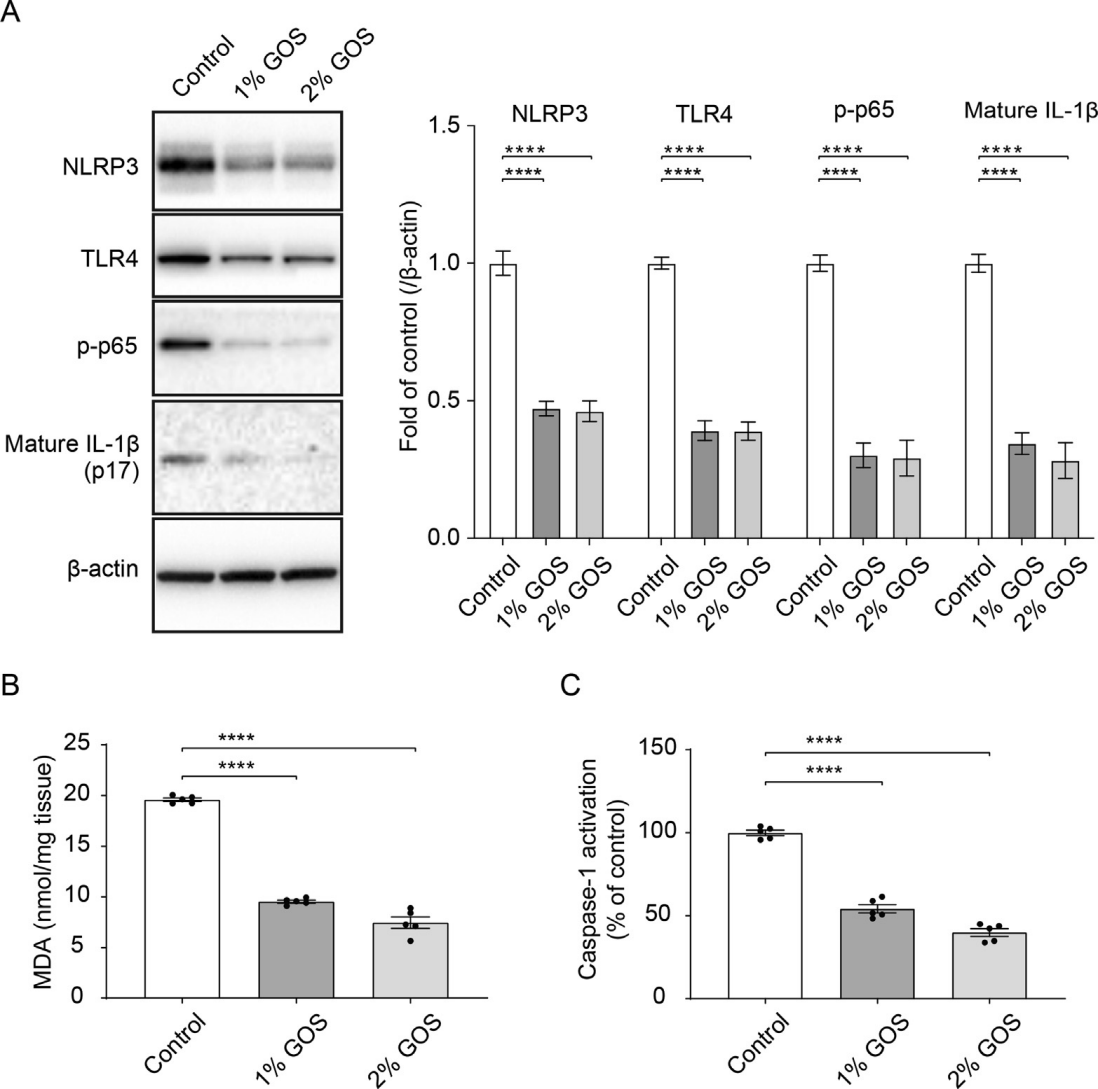
**Inhibition of the activation of NLPR3 inflammasome by GOS *in vivo*.** Calf bronchial mucosal tissue of control, 1% GOS and 2% GOS groups was collected at week 8. (**A**) Expression of NLRP3, TLR4 and IL-1β and phosphorylation of NF-κB p65 were determined by western blotting (n = 15, 5 calves/group) (original blots are depicted in supplementary Figure S8A). (**B-C**) MDA release and caspase-1 activation were assessed in control, 1% GOS and 2% GOS groups (n = 15, 5 calves/group). Each dot represents one calf. ****P < 0.0001 (GOS treatments vs control group). Data are presented as means ± SEM. GOS = galacto-oligosaccharides; IL = interleukin; MDA = malondialdehyde; NLRP3 = NLR family pyrin domain containing 3; NF-κB = nuclear factor kappa B; TLR4 = Toll-like receptor 4.

The invasion of and damage to the bronchial mucosa by *M. haemolytica* may promote the production of inflammation (e.g., IL-1β release) and the formation of infection foci [1], [5]. The extent of NLRP3 inflammasome activation after GOS intervention was studied in the bronchial mucosal tissue. GOS significantly reduced the phosphorylation of NF-κB p65 and the expression of NLRP3, TLR4, and IL-1β (Fig. 3A) in mucosal tissue. A decreased release of MDA and activation of caspase-1 were observed after GOS intervention (Fig. 3B and C).

### Inhibition of *M. haemolytica*-induced release of cytokines and chemokines in primary bronchial epithelial cells by GOS

Epithelial cells are one of the main cell types present in the bronchial mucosa and are the first line of defense against the invasion of pathogens [3], [6]. To unravel the mechanism of GOS inhibiting *M. haemolytica*-induced inflammation, primary epithelial cells near the RCrL of healthy lungs were collected and cultured. An *ex vivo* infection model with PBECs stimulated by *M. haemolytica,* an important pathogen involved in lung infections in the present *in vivo* study, was developed [18].

**Fig. 4:**
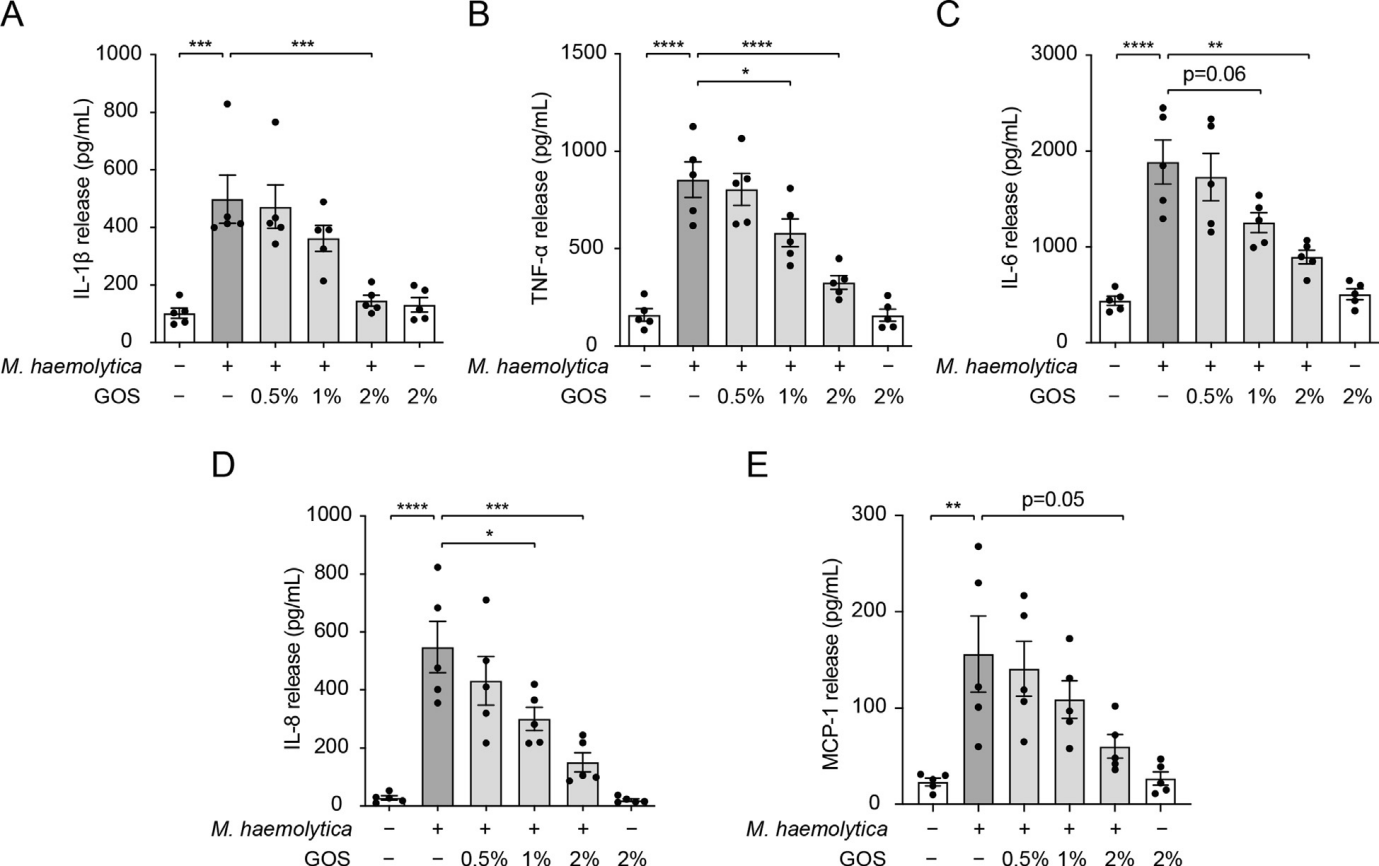
**Inhibition of *M. haemolytica*-induced release of cytokines and chemokines in primary bronchial epithelial cells by GOS.** PBECs were incubated with *M. haemolytica* (1 × 10^5^ CFU/mL) for 24 h with or without 24 h pretreatment with GOS. (**A-E**) IL-1β (**A**), TNF-α (**B**), IL-6 (**C**), IL-8 (**D**) and MCP-1 (**E**) release were measured in the supernatants of PBECs. *P < 0.05; **P < 0.01; ***P < 0.001; ****P < 0.0001. Data are presented as means ± SEM. All data shown are representative of at least five independent experiments (n = 5 donor calves). GOS = galacto-oligosaccharides; IL = interleukin; MCP = Monocyte chemoattractant protein; PBECs = primary bronchial epithelial cells; TNF = tumor necrosis factor.

In the present study, *M. haemolytica* induced a significant release of different cytokines and chemokines in PBECs, including IL-1β, TNF-α, IL-6, IL-8 and MCP-1 (Fig. 4). Interestingly, pretreatment with GOS significantly lowered the *M. haemolytica*-induced release of IL-1β, TNF-α, IL-6, and IL-8 and tended to reduce the release of MCP-1 (Fig. 4), while did not affect the cellular survival (MTT assay) and lactate dehydrogenase (LDH) release (supplementary Figure S2). In addition, GOS alone did not affect the cytokine and chemokine release in PBECs (Fig. 4).

### Inhibition of *M. haemolytica*-induced activation of NLPR3 inflammasome in primary bronchial epithelial cells by GOS

**Fig. 5:**
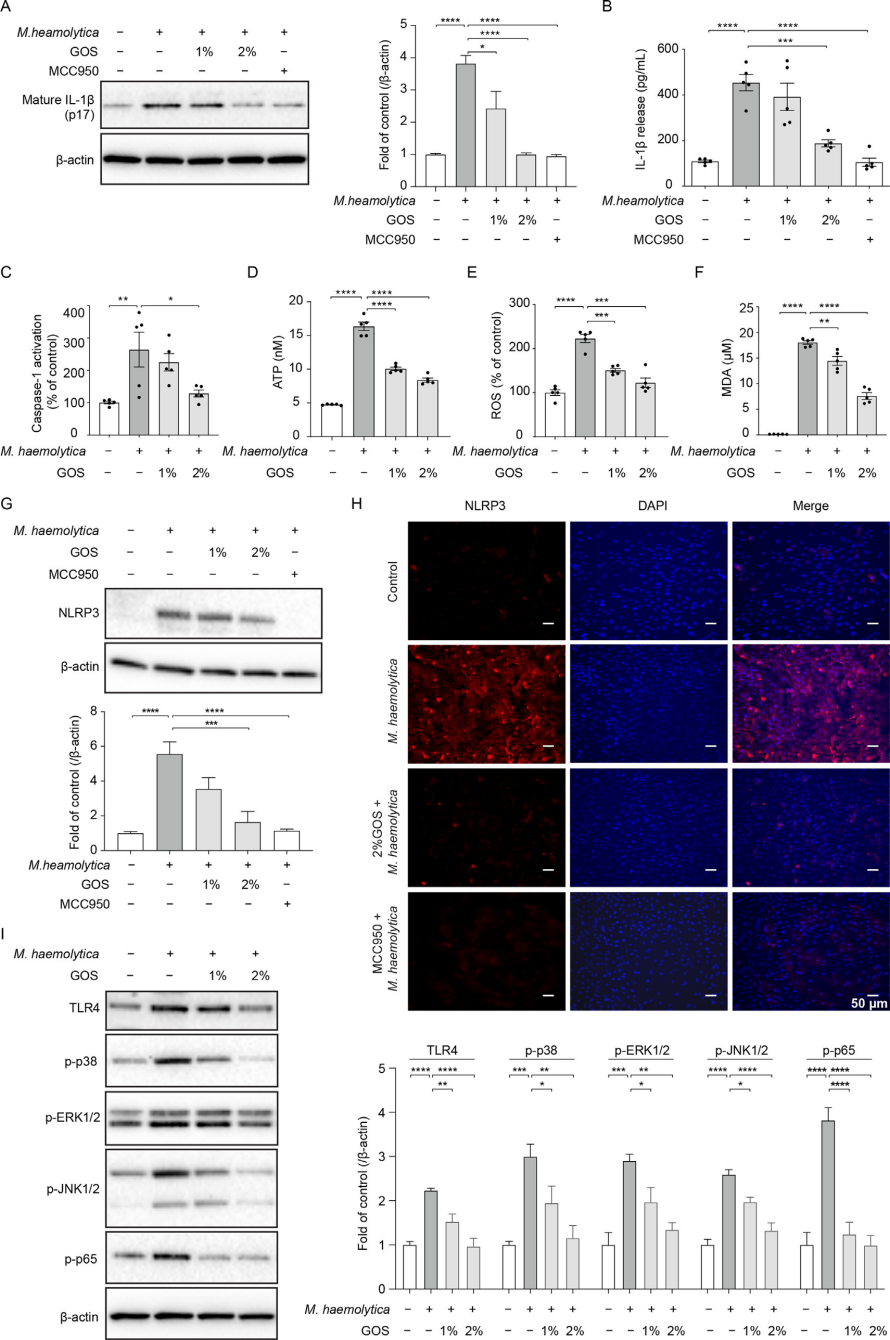
**Inhibition of *M. haemolytica*-induced activation of NLPR3 inflammasome in primary bronchial epithelial cells by GOS.** PBECs were incubated with *M. haemolytica* (1 × 10^5^ CFU/mL) for 24 h with or without GOS (24 h) or MCC950 (6 h) pretreatment. (**A-B**) Expression of mature IL-1β in cell lysates and release of IL-1β in the supernatants were examined and data were shown as a fold of control or absolute amount of IL-1β (original blots are depicted in supplementary Figure S8B). (**C-F**) Caspase-1 activation and ATP, ROS and MDA production in PBECs were assessed and data were shown as a percentage of control or absolute amount. (**G**) Expression of NLRP3 was determined by immunoblot and results were shown as a fold of control (original blots are depicted in supplementary Figure S8B). (**H**) PBECs were stained for NLRP3 (red), followed by counterstaining with DAPI (blue). (**I**) Expression of TLR4 and phosphorylation of p38, ERK1/2, JNK1/2 MAPK and NF-κB p65 were determined by immunoblot and results were shown as a fold of control (original blots are depicted in supplementary Figure S8C). *P < 0.05; **P < 0.01; ***P < 0.001; ****P < 0.0001. Data are presented as means ± SEM. All data shown are representative of at least five independent experiments (n = 5 donor calves). ATP = adenosine triphosphate; GOS = galacto-oligosaccharides; IL = interleukin; MDA = malondialdehyde; MAPK = mitogen-activated protein kinase; NLRP3 = NLR family pyrin domain containing 3; NF-κB = nuclear factor kappa B; PBECs = primary bronchial epithelial cells; ROS = reactive oxygen species; TLR4 = Toll-like receptor 4.

To investigate the activation of NLPR3 inflammasome *in vitro*, IL-1β and NLRP3 expression, mitochondrial function and caspase-1 activation were examined. Western blotting of cell lysates and ELISA data showed that 24 h pretreatment with GOS significantly inhibited the *M. haemolytica*-induced expression and release of mature IL-1β, respectively, which was also observed after preincubation with NLRP3 inflammasome inhibitor MCC950 (Fig. 5A and B). Furthermore, pretreatment with GOS inhibited the *M. haemolytica*-induced activation of caspase-1 and production of ATP, ROS, and MDA (Fig. 5C-F). Remarkably, by pretreating PBECs with ROS inhibitors, acetylcysteine (NAC) significantly decreased *M. haemolytica*-induced IL-1β release (supplementary Figure S3).

In addition, GOS significantly inhibited the *M. haemolytica*-induced expression of NLRP3 as measured by western blotting and immunofluorescence staining, which was comparable to the inhibitory effect of MCC950 (Fig. 5G and H). Moreover, GOS also significantly decreased the expression of TLR4, and the phosphorylation of p38, ERK1/2, JNK1/2 MAPK and NF-κB p65 (Fig. 5I).

### Inhibition of LPS-induced NLPR3 inflammasome activation in both bovine primary bronchial epithelial cells and human lung epithelial cells by GOS

*M. haemolytica* colonize and invade the bronchial mucosa with the help of released LPS [5]. Host cells prime the formation of NLRP3 inflammasome through recognizing LPS released by pathogens [1]. Here, GOS on LPS-triggered activation of NLRP3 inflammasome was investigated.

**Fig. 6:**
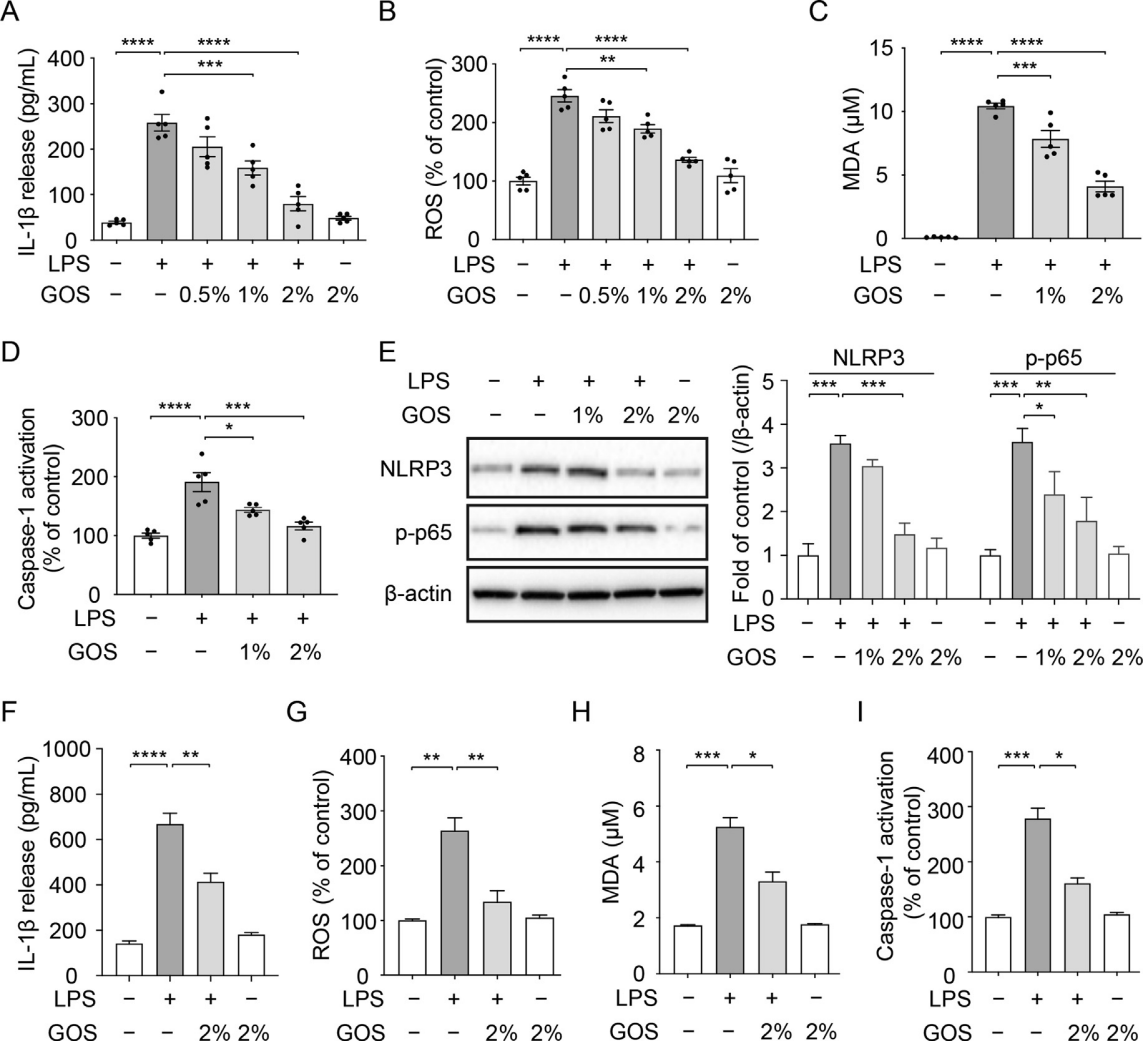
**Inhibition of LPS-induced NLPR3 inflammasome activation in both bovine primary bronchial epithelial cells and human lung epithelial cells by GOS.** PBECs or A549 cells were treated with LPS for 24 h with or without 24 h pretreatment with GOS. (**A**) IL-1β release was measured in the supernatants of PBECs. (**B**-**C**) Mitochondrial ROS and MDA production were assessed in PBECs. (**D**) Caspase-1 activation was examined in PBECs. (**E**) Expression of NLRP3 and phosphorylation of NF-κB p65 were determined in PBECs and results were shown as a fold of control (original blots are depicted in supplementary Figure S8D). (**F**) IL-1β release was measured in the supernatants of A549 cells. (**G**-**H**) Mitochondrial ROS and MDA production were assessed in A549 cells. (**I**) Caspase-1 activation was examined in A549 cells. *P < 0.05; **P < 0.01; ***P < 0.001; ****P < 0.0001. Data are presented as means ± SEM. All data shown are representative of at least five independent experiments (n = 5 donor calves or cell generations). GOS = galacto-oligosaccharides; IL = interleukin; LPS = lipopolysaccharides; MDA = malondialdehyde; NLRP3 = NLR family pyrin domain containing 3; NF-κB = nuclear factor kappa B; PBECs = primary bronchial epithelial cells; ROS = reactive oxygen species.

In the present study, LPS exposure resulted in a significant release of IL-1β and activation of caspase-1, which was facilitated by the production of ROS and MDA (Fig. 6A-D) in PBECs. In contrast, pretreatment with GOS significantly inhibited the LPS-induced release of IL-1β and activation of caspase-1, as well as the production of ROS and MDA (Fig. 6A-D), while GOS alone did not affect the IL-1β release and ROS production. The ROS inhibitor (NAC) was also able to significantly inhibit the LPS-induced IL-1β release (supplementary Figure S3). Furthermore, GOS were also effective in inhibiting the ROS production in rotenone stimulated PBECs (positive control) (supplementary Figure S4).

In addition, the increased phosphorylation pattern of NF-κB p65 and expression pattern of NLRP3 showed priming of NLRP3 inflammasome in LPS-treated PBECs (Fig. 6E). Pretreatment with GOS significantly decreased the NF-κB p65 phosphorylation and the NLRP3 expression, while GOS alone did not affect these expression patterns (Fig. 6E). Moreover, LPS with or without GOS pretreatment and GOS alone did not affect the cellular survival and LDH release in PBECs (supplementary Figure S5).

To verify the key findings obtained with bovine cells, human lung epithelial cells (A549) were preincubated with GOS and stimulated with LPS for 24 h, as described for PBECs. GOS preincubation decreased NLRP3 inflammasome activation induced by LPS as monitored by analyzing IL-1β release, ROS and MDA production and caspase-1 activation in human A549 cells (Fig. 6F-I).

### Inhibition of leukotoxin A-induced production of ATP in primary bronchial epithelial cells by GOS

Similar to LPS, leukotoxin is also responsible for lung inflammation caused by *M. haemolytica* [5]. Here, IL-1β and ATP production in PBECs after exposure to leukotoxin A (secreted by *M. haemolytica*) with or without 24 h GOS pretreatment was evaluated. Despite leukotoxin A-exposed PBECs did not show a significant increase in IL-1β release within 24 h stimulation, a significant increase in ATP production was observed after 6 h (supplementary Figure S6A and C). No cytotoxic effects of leukotoxin A on PBECs were observed till 12 h incubation (supplementary Figure S6B). The leukotoxin A-induced ATP production (after 12 h) was inhibited by preincubation with GOS (supplementary Figure S6D). The inhibition of ATP production by GOS was also observed in *M. haemolytica*-treated PBECs, while GOS treatment alone did not affect ATP production (supplementary Figure S6E).

### Inhibition of ATP-induced NLPR3 inflammasome activation in both bovine primary bronchial epithelial cells and human lung epithelial cells by GOS

**Fig. 7:**
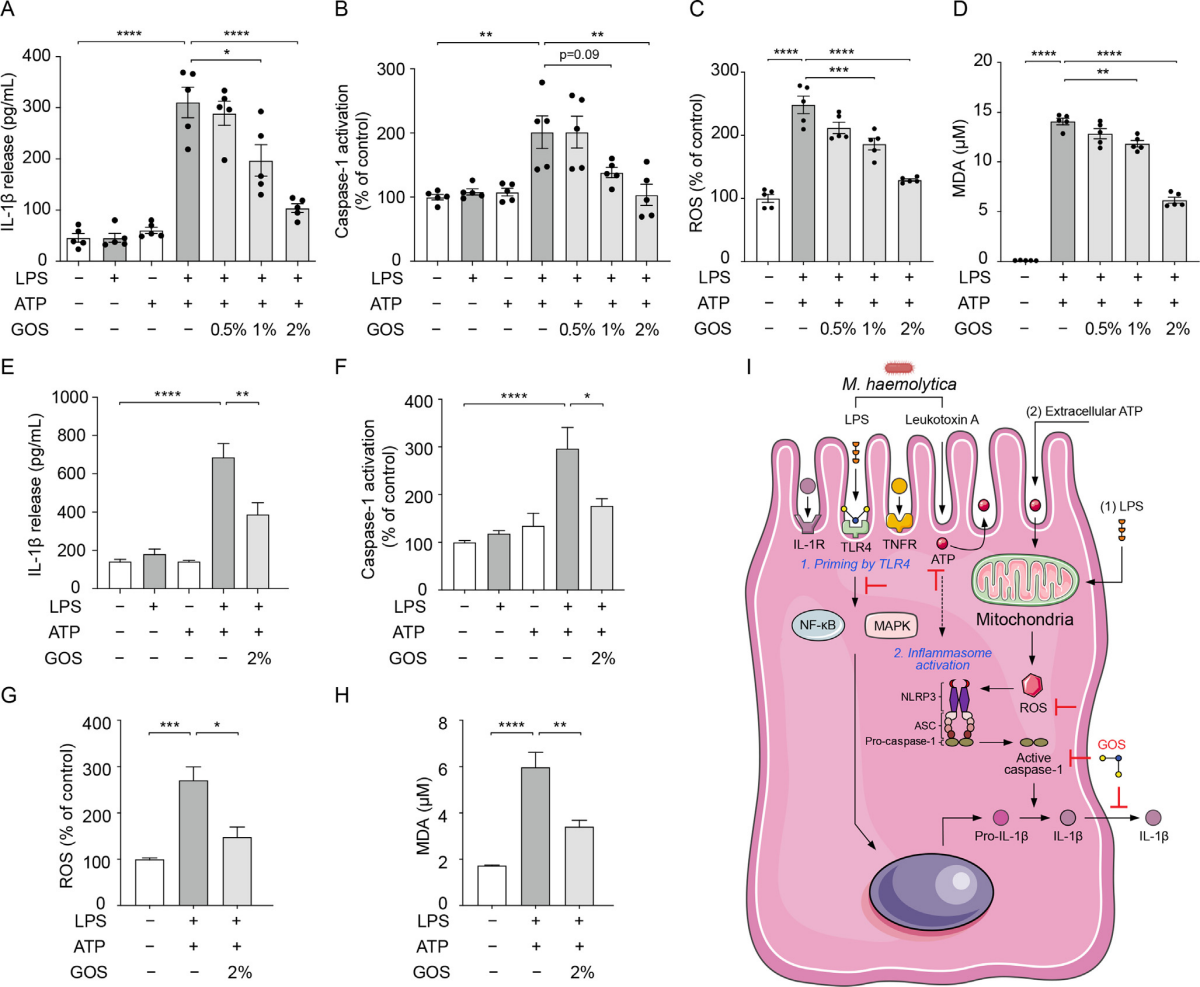
**Inhibition of ATP-induced NLPR3 inflammasome activation in both bovine primary bronchial epithelial cells and human lung epithelial cells by GOS.** PBECs or A549 cells were treated with or without GOS pretreatment (24 h) prior to the stimulation with LPS (6 h) or ATP (0.5 h) or LPS + ATP (6 h + 0.5 h). (**A**) IL-1β release was measured in the supernatants of PBECs. (**B**) Caspase-1 activation was examined in PBECs. (**C**-**D**) Mitochondrial ROS and MDA production were assessed in PBECs. (**E**) IL-1β release was measured in the supernatants of A549 cells. (**F**) Caspase-1 activation was examined in A549 cells. (**G**-**H**) Mitochondrial ROS and MDA production were assessed in A549 cells. (**I**) Pathogenesis of *M. haemolytica*-induced inflammation and postulated underlying mechanism of GOS leading to inhibition of NLRP3 inflammasome. GOS supplementation can inhibit the activation of NLRP3 inflammasome possibly by downregulating the “TLR4/NF-κB and secondarily MAPK” signaling pathway and reducing the production of ATP and mitochondrial ROS, thereby suppressing caspase-1 activation and preventing lung inflammation (e.g., mature IL-1β release). *P < 0.05; **P < 0.01; ***P < 0.001; ****P < 0.0001. Data are presented as means ± SEM. All data shown are representative of at least five independent experiments (n = 5 donor calves or cell generations). ASC = adaptor protein apoptosis-associated speck-like containing a caspase recruitment domain; ATP = adenosine triphosphate; GOS = galacto-oligosaccharides; IL = interleukin; LPS = lipopolysaccharides; MDA = malondialdehyde; MAPK = mitogen-activated protein kinase; NF-κB = nuclear factor kappa B; NLRP3 = NLR family pyrin domain containing 3; ROS = reactive oxygen species; TNF = tumor necrosis factor; TLR4 = Toll-like receptor 4.

Although leukotoxin A failed to promote IL-1β release within 24 h in PBECs, it induced ATP production within 6 h. ATP, as an endogenous danger signal, has been reported to trigger the activation of NLRP3 inflammasome via the induction of K^+^ efflux (signal 2) [1]. Here, the IL-1β release and caspase-1 activation in PBECs increased after LPS + ATP (6 h + 0.5 h) stimulation, although LPS (6 h) or ATP (0.5 h) alone could not significantly increase IL-1β release (Fig. 7A and B). Therefore, the effect of GOS on LPS + ATP-induced activation of NLRP3 inflammasome was investigated.

Interestingly, GOS displayed a significant decrease in IL-1β release, caspase-1 activation, and ROS and MDA production in PBECs exposed to LPS + ATP (Fig. 7A-D). In addition, the ROS inhibitor, NAC, significantly inhibited LPS + ATP-induced IL-1β release (supplementary Figure S3).

To verify the findings obtained with bovine cells, human A549 cells were preincubated with GOS for 24 h and stimulated with LPS + ATP, as described for PBECs. GOS preincubation decreased NLRP3 inflammasome activation induced by LPS + ATP as monitored by analyzing IL-1β release, caspase-1 activation and ROS and MDA production in human A549 cells (Fig. 7E-H).

## Discussion

Altogether, this study indicated that dietary GOS can reduce airway and systemic inflammation by restoring the immune imbalance as well as decreasing the NLRP3 inflammasome activation at least during the early stages of lung infection. Direct anti-inflammatory and anti-oxidative properties of GOS on lung cells are possibly involved as well.

Lung epithelial cells can not only form a physical barrier, but also play a central role in the recognition of pathogens and recruitment of immune cells [6]. An increase in neutrophil numbers, a decrease in macrophage numbers and production of cytokines and chemokines were observed in BALF of control calves at week 5 compared to week 1, suggesting the presence of lung infections/inflammation. These changes of immune cell composition in BALF/lungs may be partly mediated by the release of pro-inflammatory mediators from lung epithelial cells, for example, IL-8 may mediate the recruitment of neutrophils, and TNF-α and IL-1β may contribute to the activation of macrophages [19]. However, in natural exposure or inoculated infection models, clinically healthy or asymptomatic individuals might be present [20], [21], which is also a common observation in human respiratory infections. This could be the reason why the effect of GOS on BALF composition and clinical scores might still be underestimated. In addition, the insensitivity of clinical scores to the diagnosis of (subclinical) lung infections may also lead to contrasting results as compared to the measurements of cell composition and cytokine/chemokine levels in BALF/blood [22]. Furthermore, the immunomodulatory effects of GOS occurred during week 5 and seemed to disappear during week 7, which might be related to (1) the group antimicrobial treatments for all calves based on clinical scores at week 6 and/or (2) the innate immune system activation (increased BALF neutrophils and blood leukocytes) during week 5/6, which contributes to partly eliminating/phagocytosing the pathogens in the lungs.

Interestingly, up to 80% of calves are positive for *M. haemolytica* in BALF and bronchial mucosa at week 5 and 8, respectively. It has recently been reported that outbreaks of *M. haemolytica* infections increased in Dutch calves [23]. Although the presence of other opportunistic pathogens, such as *Pasteurella multocida* was not investigated in our study, substantial evidence is provided to indicate that *M. haemolytica* might be one of the pathogens involved in the present lung infections. LPS plays a critical role in the pathogenesis of *M. haemolytica*-induced pneumonia, especially in the promotion of airway inflammation [5]. Here, the presence of *M. haemolytica* released LPS in BALF and blood might be the cause of airway and systemic inflammation observed in lung infections, especially related to the IL-1β release caused by the NLRP3 inflammasome activation.

Oxidative stress-related NLRP3 inflammasome may play a central role in several inflammatory conditions in cows, while antioxidant supplementation during the peripartum period is beneficial for cow’s health [24]. Others showed that an oxidative burst is caused during the adhesion of *M. haemolytica* to bovine neutrophils [25], and increased oxidative stress, as measured by MDA levels, was detected in the serum of goats infected with *M. haemolytica* [26]. In the present study, increased lipid peroxidation was detected in the blood and bronchial mucosa in control calves, which could be due to the invasion of *M. haemolytica* and released virulence factors (e.g., LPS, leukotoxin) [5].

TLR4 on the airway epithelial cells mainly senses bacterial LPS and induces recognition to many Gram-negative pathogens [6]. In our study, *M. haemolytica* can release LPS to activate the “TLR4/NF-κB” pathway in PBECs, indicating the initiation of NLRP3 inflammasome activation (signal 1). A study in mice showed that inhalation of LPS induces an increase in TLR4 expression in bronchial epithelium and macrophages within 24 h [27]. In addition, the activation of NLRP3/ASC inflammasome by the respiratory syncytial virus in human lung epithelial cells is primed by TLR4 [28], supporting our findings that *M. haemolytica* active NLRP3 inflammasome via the recognition of released LPS by TLR4 in PBECs.

Notably, the generation of ROS/ATP caused by *M. haemolytica* or its virulence factors (LPS and leukotoxin) results in rapid activation of NLRP3 inflammasome in PBECs (signal 2). Studies in human lung epithelial cells showed that LPS exposure for 24 h resulted in strongly elevated ROS levels accompanied by mitochondrial dysfunction [29], which is in line with our data. It is possible that (long-term) LPS-induced ROS triggers the activation of NLRP3 inflammasome, which is also observed in our previous study where LPS and cigarette smoke activate NLRP3 inflammasome and induce IL-1β release in human bronchial epithelial cells within 16 h [30]. In line with these findings, we observed that NAC can reverse the IL-1β release caused by LPS exposure. Furthermore, other studies showed that exposure to extracellular ATP resulted in increased ROS production in human intestinal or gingival epithelial cells, which might be related to the activation of P2X7 receptors contributing to autophagy [31] or microbial infection [32]. Although in our study leukotoxin A failed to induce IL-1β release within 24 h, it did cause rapid ATP release after 6 h exposure to PBECs, which might trigger the (short-term) LPS-primed NLRP3 inflammasome activation.

NLRP3 inflammasome might be activated by ATP-dependent and ATP-independent mechanisms in PBECs stimulated with *M. haemolytica* (Fig. 7I). 1) Long-term LPS exposure (24 h) activates the “TLR4/NF-κB” pathway and increases mitochondrial ROS, leading to NLRP3 inflammasome activation accompanied by IL-1β release. 2) Short-term LPS exposure (6 h) needs an additional trigger, such as extracellular ATP, to initiate inflammasome activation.

Here, we reported for the first time that GOS have the potential to decrease IL-1β release via targeting/inhibiting NLRP3 inflammasome during a lung infection. In addition, the reduction in the release of other proinflammatory mediators induced by GOS, such as TNF-α, IL-6, and IL-8, were observed *in vivo* and *ex vivo*.

NDOs may indirectly exert anti-oxidative effects by regulating the gut microbiota [33]. An *in vivo* study showed a reduction in renal injury, which is probably the result of a decrease in blood oxidative stress induced by GOS-mediated microbiota changes [34]. Although mechanisms remained unclear, *in vitro* studies showed that *Lactobacillus* spp. and *Bifidobacterium* spp. fermented by several NDOs cause oxygen-free radical elimination and lipid peroxidation inhibition [12], [35]. Although not investigated in the current study, it might be possible that changes in gut microbiota induced by dietary GOS contribute to the decreased MDA concentrations, NLRP3 inflammasome activation and corresponding mediators in calves.

In addition, increasing evidence showed that oligosaccharides are absorbed into the systemic circulation after oral administration [36], [37]. After oral ingestion of breast milk, about 1% of human milk oligosaccharides (HMOs) were absorbed in the blood circulation of infants [38], [39]. Eiwegger *et al.* showed a 14% uptake of GOS across the intestinal epithelial layer within 90 min incubation *in vitro* [39]. Our previous study reported that GOS were detected in blood serum and urine of piglets after feeding 0.8% GOS once per day [38]. These studies suggest that GOS might reach the lungs (bronchus) through systemic circulation, resulting in direct inhibition of NLRP3 inflammasome and inflammation. We did not investigate the *in vivo* fate of GOS in the calves used in this study, therefore the exact concentration of GOS or its potential intermediates in the lungs (local) and circulation (systemic) of calves/ruminants is not available and it warrants further research.

No significant dose-dependent effects of 1% and 2% GOS on TLR4 and NLRP3 inflammasome activation were observed in calves. To confirm that there is no dose-dependency, more than 2 dosages of GOS (and/or a larger range of GOS dosages) are needed in the future. Moreover, 1% GOS seemed to show more optimal effects on regulating immune cell composition, like the percentages of macrophages and neutrophils in BALF compared to 2% GOS. Ingestion and fermentation of higher GOS dosages might be related to the increase in stool frequency and changes in stool consistency [40], which might decrease the absorption of nutrients, such as GOS, especially, in infected calves.

Interestingly, in addition to inhibiting the adhesion of pathogens to host epithelial cells, GOS have been found to act as TLR4 ligands to regulate host immune function, which could affect phosphorylation of NF-κB and production of cytokines and chemokines [12], [41]. Comparable to LPS, GOS might competitively bind to TLR4 of the bronchial epithelium, attenuating proinflammatory signaling (i.e., “TLR4/NF-κB and secondarily MAPK” pathway) and the priming of NLRP3 inflammasome, thereby reducing the release of cytokines. HMOs have been reported to inhibit the release of IL-8 and the phosphorylation of ERK and NF-κB caused by *E. coli* invasion of intestinal epithelial cells, which may be due to the reduction of CD4 binding to TLR4 [42]. Another *in vitro* study pointed out that chitosan-oligosaccharides (COS) can inhibit the activation of MAPK and NF-κB and the production of IL-1β and NO in LPS-treated RAW 264.7 cells, possibly because COS suppress the binding of LPS to the TLR4/MD-2 receptor complex [43]. In addition to possible direct effects on TLR4 functions, NDOs can interfere with TLR4-mediated proinflammatory signals by directly regulating the host kinase network [44], which is in line with our observations that GOS inhibited the phosphorylation of NF-κB p65 and MAPKs.

Remarkedly, it is thought that NDOs can neutralize or interfere with bacterial toxins [12], as well as participate in ROS scavenging and peroxidase reduction directly [33]. Our *ex vivo* observations indicated that GOS can reduce *M. haemolytica* and leukotoxin A-induced ATP production as well as can lower *M. haemolytica* and LPS-induced mitochondrial peroxidation. Moreover, GOS prevented rotenone (ROS agonist) -induced mitochondrial ROS production and the antioxidant NAC lowered *M. haemolytica,* LPS and ATP-induced IL-1β release in PBECs, suggesting that the anti-oxidative effect of GOS might be one of the mechanisms for the reduction of NLRP3 inflammasome activation and inhibition of the proinflammatory mediator release. Furthermore, both GOS and NLRP3 inflammasome inhibitor (MCC950) inhibited *M. haemolytica*-induced NLRP3 protein expression and IL-1β release in PBECs. To confirm the essential role of NLRP3 inflammasome in the anti-inflammatory effect of GOS, the knock-down and/or over-expression of NLRP3 protein in PBECs and human lung epithelial cells need to be investigated in the future.

The postulated underlying mechanism of GOS leading to inhibition of NLRP3 inflammasome is summarized in Fig. 7I. GOS supplementation can inhibit the activation of NLRP3 inflammasome possibly by downregulating the “TLR4/NF-κB and secondarily MAPK” signaling pathway and reducing the production of ATP and mitochondrial ROS, thereby suppressing caspase-1 activation and preventing lung inflammation (e.g., mature IL-1β release).

After the observation that GOS exhibit anti-inflammatory properties in bovine PBECs, a human lung epithelial (A549) cell line was used to confirm the anti-inflammatory activity in human airway epithelial cells. Although the A549 cell line is a lung cancer-derived cell line, it has a proper NLRP3 inflammasome activation and pro-inflammatory response in reaction to the stimulation of LPS and pathogenic stimulations [45], [46]. The *in vitro* data from human A549 cells are in line with the data from bovine PBECs, showing the promising possibility of supplementing GOS in preventing human respiratory inflammation/infection. In addition, the inhibition of IL-1β release by GOS was also observed in a SV40-immortalized human bronchial epithelial cell (16HBE) line stimulated with LPS and LPS + ATP (supplementary Figure S7), which is in line with the data observed with A549 cells and PBECs. Due to the limitation of (cancer-derived) cell lines, validation of the anti-inflammatory effects of GOS in human primary lung epithelial cells (from childhood) could be an interesting subject for future research.

## Conclusion

In conclusion, NLRP3 inflammasome activation was observed in the airways of calves with lung infections, which may contribute to the elevated lung inflammation *in vivo* and may be associated with the activation of NLRP3 inflammasome in bronchial epithelial cells caused by *M. haemolytica* and its released LPS/leukotoxin. For the first time, the observed inhibitory effect of GOS on NLRP3 inflammasome activation brings us one step closer to the understanding of the anti-inflammatory mechanism of GOS, which could be important for their beneficial effect on respiratory infections.

## Materials and methods

### Ethics statement

This experiment was conducted under the Dutch Law on Animal Experiments in accordance with EU Directive 2010/63 at the research facilities of the VanDrie Group (Scherpenzeel, The Netherlands) and was approved by the Animal Care and Use Committee of Wageningen University (AVD1040020185828, Wageningen, The Netherlands).

### Animal experiment design

The experiment consisted of 2 periods, experimental period, and growing period. During these periods, all calves were naturally exposed to pathogens in the environment. Period 1 (experimental period) started when 150 male Holstein Friesian calves arrived at the experimental facilities (∼18 days of age) and lasted from experimental week 1 till 8 in which GOS treatments were applied and most of the measurements were conducted on individual calves. At the end of period 1, 10 calves of each group were sacrificed, and bronchial mucosal tissue was collected. Period 2 lasted from experimental week 9 to slaughter at week 27 and lung scores were performed in the slaughterhouse. In period 2, no oligosaccharide treatments were applied, and all calves received the same diet. Measurements and analyses were performed for all calves or for a sub-set of calves in a blinded manner. The sub-set of calves included 2 calves per pen and 20 calves per group and was selected on body weight at arrival, closest to the average body weight of all calves at arrival.

The *in vivo* study described in this article was part of a large calf trial, including a control group, 1% GOS group, 2% GOS group and 3 other groups with different (dietary) interventions [48]. In accordance with the purpose of this study, investigating the effect of oral GOS on lung infection, we reported the results of the analyses of the control and GOS groups.

### Experimental diet

In period 1, 150 male Holstein Friesian calves (43.3 ± 0.26 kg, means ± SEM) of German origin were used and assigned randomly to 3 groups supplying with calf milk replacer (MR) with or without GOS (Vivinal GOS syrup, FrieslandCampina Ingredients, The Netherlands) twice a day. The detailed composition of GOS is summarized in Fig. 1B. The MR mainly contained 527 g/kg whey powder, 35 g/kg lactose, 52 g/kg delactosed whey powder, 50 g/kg whey protein concentrate, 60 g/kg soy protein concentrate, 50 g/kg soluble wheat protein, 3 g/kg pea fiber, 179.4 g/kg fat sources, 9.7 g/kg calcium formate, 2 g/kg citric acid, 4 g/kg sodium bicarbonate, 3.5 g/kg mono ammonium phosphate, 9.8 g/kg lysine, 2.4 g/kg methionine, 1.3 g/kg threonine, 0.2 g/kg aroma and 10 g/kg premix. GOS administered via the MR were included at the expense of lactose, corrected for the purity and DM of the GOS products used. Group 1 as a control group included 50 calves and received MR without GOS. Group 2 and 3 included 50 calves and received MR containing 1% or 2% GOS, respectively.

### BALF sampling and phenotyping

BALF was obtained by use of a technique adapted from Caldow *et al.* [47]. A sterilized 100 cm BAL catheter was inserted through a naris and blindly guided through the nasal passage into the trachea until the end was wedged in a bronchus. Once wedged in the appropriate location, a syringe was connected to the catheter and a total of 30 mL sterile saline (37 °C) solution was slowly infused and immediately aspirated back into the syringe after each infusion. BALF (17.7 ± 0.4 mL) was obtained from each calf and stored in a 50 mL tube on ice until further processing in the lab the same day.

Thereafter, BALF was filtered by passing through a 70 μm cell strainer (Corning, NY) to remove debris. To obtain cell pellets and perform cell counts, BALF suspension was centrifuged (5 min, 400 × *g* at 4 °C) and the remaining pellet was re-suspended in 1 mL cold fetal bovine serum (FBS; 4 °C). After centrifugation, the supernatant was aliquoted into 1.5 mL tubes and stored at −80 °C for further analysis. Cell number was determined by automatically counting in a Cellometer Bright Field cell counter (Nexcelom Bioscience, Lawrence, MA). For differential BALF cell counts, 0.5 × 10^6^ BALF cells were used to make cytospins stained with Diff-Quick (Medion Diagnostics, Medion Diagnostics International Inc., Miami, FL) and a minimum of 400 cells were counted.

### Identification of *M. haemolytica* in BALF and bronchial mucosal tissue

DNA was extracted from BALF and bronchial mucosal tissue using PureLink Genomic DNA Mini Kit (Invitrogen, Life Technologies, San Diego, CA) following the manufacturer’s instructions. Real-time PCR methods for the detection of species-specific genes for *M. haemolytica* were performed using the primers and probes of BactoReal Kit (DVEB02911, Ingenetix GmbH, Vienna, Austria). BactoReal Kit detects the 16S rDNA gene of *M. haemolytica*. A probe-specific amplification-curve at 530 nm (FAM channel) indicates the amplification of *M. haemolytica* specific DNA.

Assay mix was prepared in a 20 μL volume that contained 10 μL of DNA Reaction Mix, 3 μL PCR grade water, 5 μL extracted DNA from samples, 1 μL primer, and 1 μL probe. Negative and positive controls were replaced by PCR grade water and positive *M. haemolytica*-DNA in the same kit, respectively. Real-time PCR was conducted on a Real-Time PCR Detection System (Bio-Rad, Hercules, CA).

### Detection of *M. haemolytica*-LPS lgG in BALF and blood

*M. haemolytica*-LPS lgG levels were measured in BALF and blood according to manufacturer's instructions (BIO/K-139, Bio-X Diagnostics, Rochefort, Belgium). Negative and positive controls were provided by the same kit. Compared to the positive control, fold changes were calculated.

### Isolation and culture of PBECs

Isolation and culture of PBECs were conducted as previously described [18]. Briefly, PBECs were isolated from bovine bronchial epithelium obtained from healthy lungs of freshly slaughtered calves aged 6–8 months provided by Ekro bv (Apeldoorn, The Netherlands). After digesting of the bronchial epithelium, PBECs were collected and grown in 5% CO_2_ at 37 °C and attached to collagen-coated plates in serum-free RPMI-1640 medium for 2–3 days until reaching near-confluence (70–90%) and then replaced with RPMI-1640 medium containing 10% FBS, 1% L-glutamine, 1% MEM NEAA, and 1% penicillin–streptomycin (Sigma-Aldrich, Zwijndrecht, The Netherlands) for future culture and experiments as described before [18].

### Human lung epithelial cell (A549) culture

Human type II alveolar basal epithelial cells (A549; ATCC, Manassas, VA) were grown in Ham's F-12 K Medium (Gibco, Thermo Fisher Scientific, Waltham, MA) supplemented with 10% FBS and 1% penicillin–streptomycin (Sigma-Aldrich) in 5% CO_2_ at 37 °C.

### Bacterial growth conditions

*M. haemolytica* (isolated from infected lungs of a pneumonic calf) was kindly provided by Prof. Jos van Putten (Department of Infectious Diseases and Immunology, Utrecht University, The Netherlands). *M. haemolytica* was incubated overnight at 37 °C in 5% sheep blood agar (bioTRADING, Mijdrecht, The Netherlands).

### PBECs and A549 treatments

PBECs were cultured at a density of 1 × 10^6^ cells/mL in 96- or 6-well plates (Corning) pre-coated with collagen, fibronectin and BSA as described before [18]. After reaching near-confluence, these PBECs were pretreated with 0.5%, 1% or 2% GOS for 24 h or pretreated with MCC950 (10 μM; InvivoGen, San Diego, CA) for 6 h prior to stimulation with LPS (10 µg/mL; isolated from *E. coli O111:B4*, Sigma-Aldrich) for 6 or 24 h with or without ATP (5 mM; InvivoGen) for 0.5 h, or stimulation with leukotoxin A (10 ng/mL; Enzo Life Sciences, Bruxelles, Belgium) for 0.5, 1, 6, 12 or 24 h or stimulation with *M. haemolytica* (1 × 10^5^ CFU/mL) for 24 h, or stimulation with rotenone (10 μM; Sigma-Aldrich) for 6 h. After stimulation, supernatants were collected and stored at −20 °C until analysis.

A549 cells were cultured at a density of 0.5 × 10^5^ cells/mL in 96- or 6-well plates (Corning). After reaching near-confluence, A549 cells were pretreated with 2% GOS for 24 h prior to stimulation with LPS (10 µg/mL; *E. coli O111:B4*, Sigma-Aldrich) for 6 or 24 h with or without ATP (5 mM; InvivoGen) for 0.5 h. After stimulation, supernatants were collected and stored at −20 °C until analysis.

### Caspase-1 activation assay

Caspase-1 activity in PBECs and A549 cells was determined in 50 μL cell lysates using a commercial kit (ab39412, Abcam, Cambridge, UK) according to the manufacturer’s instructions. For the *in vivo* experiments, same weight of bronchial mucosal tissue was homogenized in lysis buffer from the kit, and 50 μL of the lysates was assayed following the manufacturer’s instructions. Assays were performed in duplicate, and averages were taken. The results were shown as the percentage of control.

### Lipid peroxidation and ROS measurements

Lipid peroxidation (malondialdehyde; MDA) in the PBECs, A549 cells, blood and bronchial mucosal tissue was measured using a commercial kit (ab118970, Abcam) according to the manufacturer’s instructions. ROS in PBECs and A549 cells were assessed using the cell-permeant probe H_2_DCFDA (MP36103, Invitrogen, Thermo Fisher Scientific) according to the manufacturer’s instructions. The level of ROS was shown as the percentage of control.

### ATP measurement

The production of ATP was measured using the ATP determination kit (A22066, Invitrogen, Thermo Fisher Scientific) following the manufacturer’s instructions. Briefly, PBECs after different treatments were resuspended and gently mixed in reaction buffer containing 1 mM DTT, 0.5 mM luciferin, and 1.25 μg/mL luciferase, and readings were taken in a luminometer (GloMax, Promega Corp., Madison, WI).

### ELISA measurement

Levels of IL-8 (Mabtech, Nacka Strand, Sweden), IL-6 (Invitrogen, Thermo Fisher Scientific), IL-1β (Invitrogen, Thermo Fisher Scientific) TNF-α (R&D Systems, Minneapolis, MN) and/or MCP-1 (Invitrogen, Thermo Fisher Scientific) in the BALF and blood of calves or in the supernatants of PBECs were determined by using ELISA kits according to manufacturer's instructions. Levels of IL-1β (BioLegend, San Diego, CA) in the supernatants of A549 and 16HBE cells after different treatments were also measured by using the ELISA kits. The absorbance was measured at 450 nm using a microplate reader (Bio-Rad).

### Western blotting

Cell lysates of PBECs and tissue lysates of calves after different treatments were prepared by adding RIPA cell lysis buffer (Thermo Fisher Scientific) containing protease and phosphatase inhibitors (Roche Applied Science, Pennsburg, Germany). Total protein content was estimated by bicinchoninic acid analysis (Pierce, Thermo Fisher Scientific) according to the manufacturer’s protocol. Samples were loaded onto polyacrylamide gradient gels (4–20% Tris-HCl, Bio-Rad) and electrotransferred onto polyvinylidene difluoride membranes (Bio-Rad). The membranes were blocked with PBS containing 0.05% Tween-20 (PBST) and 5% milk proteins for 1 h at room temperature and incubated with primary antibodies at 4 °C overnight (NLRP3, 1:1000, PA5-18118; TLR4, 1:1000, PA5-23284, Thermo Fisher Scientific; IL-1β, 1:100, MCA-1658, Bio-Rad; p-p38, 1:1000, #9215; p-ERK1/2, 1:1000, #9101; p-JNK1/2, 1:1000, #9251; p-p65, 1:1000, #3033; β-actin, 1:5000, #4970, Cell Signaling Technology, Beverly, MA), followed by washing blots in PBST. Appropriate horseradish peroxidase-coupled secondary antibodies from Dako (Agilent Technologies, Santa Clara, CA) were applied for 1 h. Membranes were incubated with ECL western blotting substrates (Bio-Rad) prior to obtaining the digital images. Digital images were acquired with the Molecular Imager (Gel DocTM XR, Bio-Rad) and analyzed with Image lab 5.0 (Bio-Rad).

### Immunofluorescence

PBECs were grown in 6-well plates as described above and detected for the NLRP3 protein using immunofluorescence. PBECs were fixed with 10% formalin (Baker, Deventer, The Netherlands) and after washing with PBS, the cells were permeabilized with PBS containing 0.1% (v/v) Triton X-100 for 5 min, followed by blocking with 5% serum in 1% (w/v) BSA/PBS for 30 min at room temperature. Thereafter, PBECs were incubated overnight with primary antibodies NLRP3 (1:50, ab4207, Abcam) followed by incubation with Alexa-Fluor conjugated secondary antibodies (Invitrogen) for 1 h at room temperature in the dark. Nuclear counterstaining was performed with DAPI containing anti-fade reagent (ready to use, Invitrogen). NLRP3 were visualized and images were taken using the Keyence BZ-9000 (KEYENCE Corporation, Osaka, Japan).

### Statistical analysis

Experimental results *in vivo* are expressed as non-transformed means ± SEM. *In vivo* data were analyzed for treatment and time effects with SAS 9.4 (SAS Institute Inc., Cary, NC), using the MIXED procedure, including time as a random statement with calf as unit. For each parameter, the covariance structure was selected based on the lowest AIC and BIC. All analyses included a random effect of pen. For leukocyte counts, the concentration/percentage at arrival (before application of the treatments) was included as a co-variable in the model. Studentized residuals of each model were checked visually on the homogeneity of variance and data were transformed if required to obtain homogeneity of variance. To evaluate differences between treatments, the contrast statement was used, and treatment differences were assessed per time-point separately. The effect of time on clinical scores was assessed with the estimate statement, using the GLIMMIX procedure with a multinomial distribution including a random pen effect. The Chi-square test was performed for the proportion of different lung lesions and the positivity of *M. haemolytica* in calves. Differences were considered significant when *P* < 0.05 and considered a trend when *P* < 0.10.

Data from *in vitro* experiments are determined by one-way ANOVA or two-way ANOVA followed by Tukey with selected comparisons as a *post hoc* test when F achieved *P* < 0.05 and there was no significant variance in homogeneity. All experimental results are expressed as means ± SEM and analyzed using the GraphPad Prism version 7.0 software (San Diego, CA). Results were considered statistically significant when *P* < 0.05 and considered a trend when *P* < 0.10.

## Compliance with Ethics Requirements

All Institutional and National Guidelines for the care and use of animals (fisheries) were followed.

### CRediT authorship contribution statement

**Yang Cai:** Conceptualization, Formal analysis, Investigation, Methodology, Visualization, Writing – original draft. **Myrthe S. Gilbert:** Formal analysis, Investigation, Project administration. **Walter J.J. Gerrits:** Conceptualization, Supervision, Funding acquisition, Writing – review & editing. **Gert Folkerts:** Conceptualization, Supervision, Funding acquisition, Writing – review & editing. **Saskia Braber:** Conceptualization, Methodology, Project administration, Supervision, Writing – review & editing.

## Declaration of Competing Interest

*The authors declare that they have no known competing financial interests or personal relationships that could have appeared to influence the work reported in this paper.*

## References

[1] Ravi Kumar S., Paudel S., Ghimire L., Bergeron S., Cai S., Zemans R.L. (2018). Emerging Roles of Inflammasomes in Acute Pneumonia. Am J Respir Crit Care Med.

[2] Ackermann M.R., Derscheid R., Roth J.A. (2010). Innate immunology of bovine respiratory disease. Vet Clin North Am Food Anim Pract.

[3] Caswell J.L. (2014). Failure of respiratory defenses in the pathogenesis of bacterial pneumonia of cattle. Vet Pathol.

[4] Bem R.A., Domachowske J.B., Rosenberg H.F. (2011). Animal models of human respiratory syncytial virus disease. Am J Physiol Lung Cell Mol Physiol.

[5] Singh K., Ritchey J.W., Confer A.W. (2011). Mannheimia haemolytica: bacterial-host interactions in bovine pneumonia. Vet Pathol.

[6] Leiva-Juarez M.M., Kolls J.K., Evans S.E. (2018). Lung epithelial cells: therapeutically inducible effectors of antimicrobial defense. Mucosal Immunol.

[7] Mariathasan S., Weiss D.S., Newton K., McBride J., O'Rourke K., Roose-Girma M. (2006). Cryopyrin activates the inflammasome in response to toxins and ATP. Nature.

[8] Witzenrath M., Pache F., Lorenz D., Koppe U., Gutbier B., Tabeling C. (2011). The NLRP3 Inflammasome Is Differentially Activated by Pneumolysin Variants and Contributes to Host Defense in Pneumococcal Pneumonia. J Immunol.

[9] Willingham S.B., Allen I.C., Bergstralh D.T., Brickey W.J., Huang M.-H., Taxman D.J. (2009). NLRP3 (NALP3, Cryopyrin) Facilitates In Vivo Caspase-1 Activation, Necrosis, and HMGB1 Release via Inflammasome-Dependent and -Independent Pathways. J Immunol.

[10] Mussatto S.I., Mancilha I.M. (2007). Non-digestible oligosaccharides: A review. Carbohydr Polym.

[11] Janbazacyabar H., van Bergenhenegouwen J., Verheijden K.A.T., Leusink-Muis T., van Helvoort A., Garssen J. (2019). Non-digestible oligosaccharides partially prevent the development of LPS-induced lung emphysema in mice. PharmaNutrition.

[12] Cai Y., Folkerts J., Folkerts G., Maurer M., Braber S. (2020). Microbiota-dependent and -independent effects of dietary fibre on human health. Br J Pharmacol.

[13] Bernard H., Desseyn J.-L., Bartke N., Kleinjans L., Stahl B., Belzer C. (2015). Dietary pectin-derived acidic oligosaccharides improve the pulmonary bacterial clearance of Pseudomonas aeruginosa lung infection in mice by modulating intestinal microbiota and immunity. J Infect Dis.

[14] Arslanoglu S., Moro G.E., Boehm G. (2007). Early supplementation of prebiotic oligosaccharides protects formula-fed infants against infections during the first 6 months of life. J Nutr.

[15] Arslanoglu, S., et al., Early dietary intervention with a mixture of prebiotic oligosaccharides reduces the incidence of allergic manifestations and infections during the first two years of life. J Nutr, 2008. **138**(6): p. 1091–5, Doi: 10.1093/jn/138.6.1091. 10.1093/jn/138.6.1091 18492839

[16] Hughes, C., et al., Galactooligosaccharide supplementation reduces stress-induced gastrointestinal dysfunction and days of cold or flu: a randomized, double-blind, controlled trial in healthy university students. American Journal of Clinical Nutrition, 2011. **93**(6): p. 1305–1311, Doi: 10.3945/ajcn.111.014126. 10.3945/ajcn.111.014126 21525194

[17] Dagleish M.P., Finlayson J., Bayne C., MacDonald S., Sales J., Hodgson J.C. (2010). Characterization and Time Course of Pulmonary Lesions in Calves after Intratracheal Infection with Pasteurella multocida A:3. J Comp Pathol.

[18] Cai Y., Varasteh S., van Putten J.P.M., Folkerts G., Braber S. (2020). Mannheimia haemolytica and lipopolysaccharide induce airway epithelial inflammatory responses in an extensively developed ex vivo calf model. Sci Rep.

[19] Vareille M., Kieninger E., Edwards M.R., Regamey N. (2011). The airway epithelium: soldier in the fight against respiratory viruses. Clin Microbiol Rev.

[20] Amat S., Alexander T.W., Holman D.B., Schwinghamer T., Timsit E., Gibbons S.M. (2020). Intranasal Bacterial Therapeutics Reduce Colonization by the Respiratory Pathogen Mannheimia haemolytica in Dairy Calves. mSystems.

[21] Van Driessche L., Valgaeren B.R., Gille L., Boyen F., Ducatelle R., Haesebrouck F. (2017). A Deep Nasopharyngeal Swab Versus Nonendoscopic Bronchoalveolar Lavage for Isolation of Bacterial Pathogens from Preweaned Calves With Respiratory Disease. J Vet Intern Med.

[22] van Leenen K., Van Driessche L., De Cremer L., Masmeijer C., Boyen F., Deprez P. (2020). Comparison of bronchoalveolar lavage fluid bacteriology and cytology in calves classified based on combined clinical scoring and lung ultrasonography. Prev Vet Med.

[23] Biesheuvel M.M., van Schaik G., Meertens N.M., Peperkamp N.H., van Engelen E., van Garderen E. (2021). Emergence of fatal Mannheimia haemolytica infections in cattle in the Netherlands. Vet J.

[24] Castillo C. (2019). Is the NLRP3 inflammasome a potential biomarker to avoid the misuse of antibiotics of dairy cows during the transition period?. Large Animal Rev.

[25] Kisiela D.I., Czuprynski C.J. (2009). Identification of Mannheimia haemolytica adhesins involved in binding to bovine bronchial epithelial cells. Infect Immun.

[26] Jarikre T.A., Taiwo J.O., Emikpe B.O., Akpavie S.O. (2019). Protective effect of intranasal peste des petits ruminants virus and bacterin vaccinations: Clinical, hematological, serological, and serum oxidative stress changes in challenged goats. Vet World.

[27] Saito T., Yamamoto T., Kazawa T., Gejyo H., Naito M. (2005). Expression of toll-like receptor 2 and 4 in lipopolysaccharide-induced lung injury in mouse. Cell Tissue Res.

[28] Triantafilou K., Kar S., Vakakis E., Kotecha S., Triantafilou M. (2013). Human respiratory syncytial virus viroporin SH: a viral recognition pathway used by the host to signal inflammasome activation. Thorax.

[29] Chuang C.-Y., Chen T.-L., Cherng Y.-G., Tai Y.-T., Chen T.-G., Chen R.-M. (2011). Lipopolysaccharide induces apoptotic insults to human alveolar epithelial A549 cells through reactive oxygen species-mediated activation of an intrinsic mitochondrion-dependent pathway. Arch Toxicol.

[30] Mortaz E., Henricks P.A.J., Kraneveld A.D., Givi M.E., Garssen J., Folkerts G. (2011). Cigarette smoke induces the release of CXCL-8 from human bronchial epithelial cells via TLRs and induction of the inflammasome. Biochimica Et Biophysica Acta-Molecular Basis of Disease.

[31] Souza C.O., Santoro G.F., Figliuolo V.R., Nanini H.F., de Souza H.S.P., Castelo-Branco M.T.L. (2012). Extracellular ATP induces cell death in human intestinal epithelial cells. Biochim Biophys Acta.

[32] Hung, S.C., et al., P2X4 assembles with P2X7 and pannexin-1 in gingival epithelial cells and modulates ATP-induced reactive oxygen species production and inflammasome activation. PLoS One, 2013. **8**(7): p. e70210, Doi: 10.1371/journal.pone.0070210. 10.1371/journal.pone.0070210 PMC3723664 23936165

[33] Van den Ende W., Peshev D., De Gara L. (2011). Disease prevention by natural antioxidants and prebiotics acting as ROS scavengers in the gastrointestinal tract. Trends Food Sci Technol.

[34] Furuse S.U., Ohse T., Jo-Watanabe A., Shigehisa A., Kawakami K., Matsuki T. (2014). Galacto-oligosaccharides attenuate renal injury with microbiota modification. Physiol Rep.

[35] Lin M.Y., Yen C.L. (1999). Inhibition of lipid peroxidation by Lactobacillus acidophilus and Bifidobacterium longum. J Agric Food Chem.

[36] Vazquez E., Santos-Fandila A., Buck R., Rueda R., Ramirez M. (2017). Major human milk oligosaccharides are absorbed into the systemic circulation after oral administration in rats. Br J Nutr.

[37] Ruhaak L.R., Stroble C., Underwood M.A., Lebrilla C.B. (2014). Detection of milk oligosaccharides in plasma of infants. Anal Bioanal Chem.

[38] Difilippo E., Bettonvil M., Willems R.(H.A.M.)., Braber S., Fink-Gremmels J., Jeurink P.V. (2015). Oligosaccharides in Urine, Blood, and Feces of Piglets Fed Milk Replacer Containing Galacto-oligosaccharides. J Agric Food Chem.

[39] Eiwegger, T., et al., *Prebiotic oligosaccharides: in vitro evidence for gastrointestinal epithelial transfer and immunomodulatory properties.* Pediatr Allergy Immunol, 2010. **21**(8): p. 1179–88, Doi: 10.1111/j.1399-3038.2010.01062.x. 10.1111/j.1399-3038.2010.01062.x 20444147

[40] Agostoni C., Axelsson I., Goulet O., Koletzko B., Michaelsen K.F., Puntis J.W.L. (2004). Prebiotic oligosaccharides in dietetic products for infants: a commentary by the ESPGHAN Committee on Nutrition. J Pediatr Gastroenterol Nutr.

[41] He Y., Lawlor N.T., Newburg D.S. (2016). Human Milk Components Modulate Toll-Like Receptor-Mediated Inflammation. Adv Nutr.

[42] He YingYing, Liu ShuBai, Kling D.E., Leone S., Lawlor N.T., Huang Y.i. (2016). The human milk oligosaccharide 2'-fucosyllactose modulates CD14 expression in human enterocytes, thereby attenuating LPS-induced inflammation. Gut.

[43] Qiao Y., Ruan Y., Xiong C., Xu Q., Wei P., Ma P. (2010). Chitosan oligosaccharides suppressant LPS binding to TLR4/MD-2 receptor complex. Carbohydr Polym.

[44] Wu R.Y., Määttänen P., Napper S., Scruten E., Li B.o., Koike Y. (2017). Non-digestible oligosaccharides directly regulate host kinome to modulate host inflammatory responses without alterations in the gut microbiota. Microbiome.

[45] Xu W.-J., Wang X.-X., Jin J.-J., Zou Q., Wu L., Lv T.-F. (2019). Inhibition of GGPPS1 attenuated LPS-induced acute lung injury and was associated with NLRP3 inflammasome suppression. Am J Physiol Lung Cell Mol Physiol.

[46] Pan P., Shen M., Yu Z., Ge W., Chen K., Tian M. (2021). SARS-CoV-2 N protein promotes NLRP3 inflammasome activation to induce hyperinflammation. Nat Commun.

[47] Caldow G. (2001). Bronchoalveolar lavage in the investigation of bovine respiratory disease. In Practice.

[48] Cai Y., Gilbert M.S., Gerrits W.J.J., Folkerts G., Braber S. (2021). Anti-inflammatory properties of fructo-oligosaccharides in a calf lung infection model and in *Mannheimia haemolytica*-infected airway epithelial cells. Nutrients.

