# Supplementary material for: Galacto-oligosaccharides alleviate lung inflammation by inhibiting NLRP3 inflammasome activation in vivo and in vitro
Source: J Adv Res. 2021 Nov 1;39:305–18. doi: 10.1016/j.jare.2021.10.013 (PMC9263649; doi:10.1016/j.jare.2021.10.013)
Supplement: Supplementary data 1 [file mmc1.pdf]

Galacto-oligosaccharides alleviate lung inflammation by inhibiting NLRP3 inflammasome activation *in vivo* and *in vitro*

Supplemental methods

Animal housing

Calves were housed in a mechanically ventilated stable throughout the experiment. The ambient lighting consisted of natural lighting plus artificial lighting from 0600 to 1800 h. Calves were housed in pens (9 m^2) containing wooden-slatted floors. In the first 6 weeks after arrival, individual housing was applied (1.2 m^2/calf) by placing stainless steel fences within the pens. After 6 weeks, the individual fencing was removed, and calves were housed in groups of 5.

Blood sampling and hematological analyses

Blood samples were collected of all calves by venipuncture in the jugular vein at arrival before the first MR feeding (baseline, week 0), and additionally at experimental week 2, 4 and 6 from 20 calves per group. Blood was collected in 9 mL and 4 mL K_2-EDTA tubes and was kept on ice for collection of plasma or kept at room temperature for analysis of leukocyte numbers the same day by fluorescence flow cytometry using a Sysmex 1800iV (Sysmex Europe GmbH, Norderstedt, Germany), respectively. Plasma was collected after centrifugation at 2,000 x *g* and 4°C for 20 min and was stored at -20°C pending further analyses.

Lung scores

Calf lungs were scored and obtained by using a scoring system adapted from Leruste *et al.* [1]. Briefly, the observer (veterinarian) visually examined each lung (cranial and ventral lobes) evaluating signs of pneumonia. Each examined lung was classified according to a 4-point scale for pneumonia from healthy lung (score 0) to severe lesions (score 3). Score 0 for healthy lungs (pale orange color with no sign of pneumonia), score 1 for minimal or mild lesions (one spot of grey-red discoloration), score 2 for moderate lesions (one larger or several small spots of grey-red discoloration with a total surface of less than 1 lobe), and score 3 for severe lesions (grey-red discoloration area of at least one full lobe and/or presence of abscesses). The results were shown as a percentage of the total calves with different severity of pneumonia.

Clinical scores

Clinical scoring was performed weekly for all calves, according to the Wisconsin calf respiratory scoring system [2], in which a score from 0 to 3 was provided for rectal temperature, coughing, nasal discharge and behavior. Clinical score was calculated as the sum of these 4 scores.

Lactate dehydrogenase (LDH) assay

PBECs were grown in 96-well plates as described above and the cytotoxic effect of GOS, LPS, leukotoxin A or *M. haemolytica* on the PBECs was evaluated by measuring LDH leakage. LDH was measured in the supernatants using the CytoTox 96 nonradioactive cytotoxicity assay kit (Promega Corp.) according to manufacturer's instructions.

Thiazolyl blue tetrazolium bromide (MTT) assay

PBECs were grown in 96-well plates as described above and the viability of cells was measured using MTT assay. MTT (Sigma-Aldrich) was dissolved at a final concentration of 0.5 mg/mL in cell culture medium. Each culture well was delicately washed with pre-warmed PBS before adding a 120 μL MTT solution. After 3h incubation (37 °C, 5% CO2), the formed formazan crystals were dissolved in 100 μL of dimethyl sulfoxide (Sigma-Aldrich) and absorbance was read at 595 nm using a microplate reader (Bio-Rad).

Human bronchial epithelial cell (16HBE) culture and treatments

Human bronchial epithelial cells (16HBE; Sigma-Aldrich) were grown in MEM (Gibco, Thermo Fisher Scientific) supplemented with 10% FBS, 1% L-glutamine and 1% penicillin–streptomycin (Sigma-Aldrich) in 5% CO2 at 37°C. 16HBE cells were cultured at a density of 0.5 x 10^5 cells/mL in 96-well plates (Corning). After reaching near-confluence, 16HBE cells were pretreated with 2% GOS for 24h prior to stimulation with LPS (10 μg/mL; *E. coli O111:B4*, Sigma-Aldrich) for 6 or 24h with or without ATP (5 mM; InvivoGen) for 0.5h. After stimulation, supernatants were collected and stored at -20 °C until analysis.

Supplemental results

Table S1. The number of positive for *M. haemolytica* in BALF of control calves.

BALF samples of control calves (N=20 calves)	Week 1	Week 3	Week 5	Week7
Detection of *M. haemolytica*-LPS IgG	0	4	16	16
p-value (vs week1)	—	0.11	<0.0001	<0.0001

BALF, broncho-alveolar lavage fluid; IgG, immunoglobulin G; LPS, lipopolysaccharide.

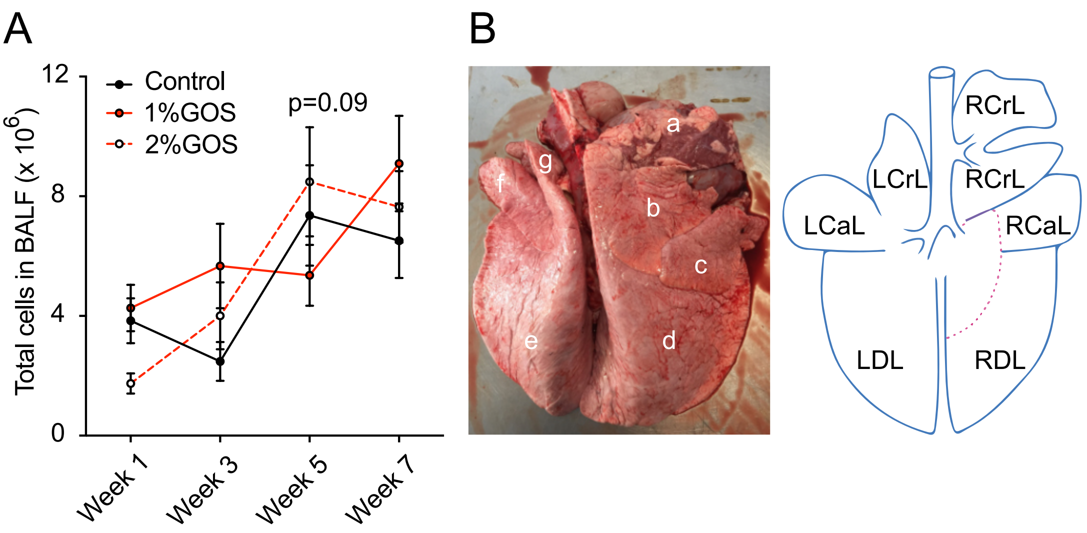

Figure S1. Effect of GOS on total cell numbers in BALF and infected calf lungs (lobes). (**A**) Number of total cells in BALF was measured at week 1, 3, 5 and 7 (n=60, 20 calves/group). (**B**) Infection foci was observed in RCrL of control calf lungs at week 8. a and b = RCrL (right cranial lobe); c = RCaL (right cardiac lobe); d = RDL (right diaphragmatic lobe); e = LDL (left diaphragmatic lobe); f = LCaL (left cardiac lobe); g = LCrL (left cranial lobe). *P*=0.09 (control week 5 vs week 1). Data are presented as means ± SEM. BALF = broncho-alveolar lavage fluid; GOS = galacto-oligosaccharides.

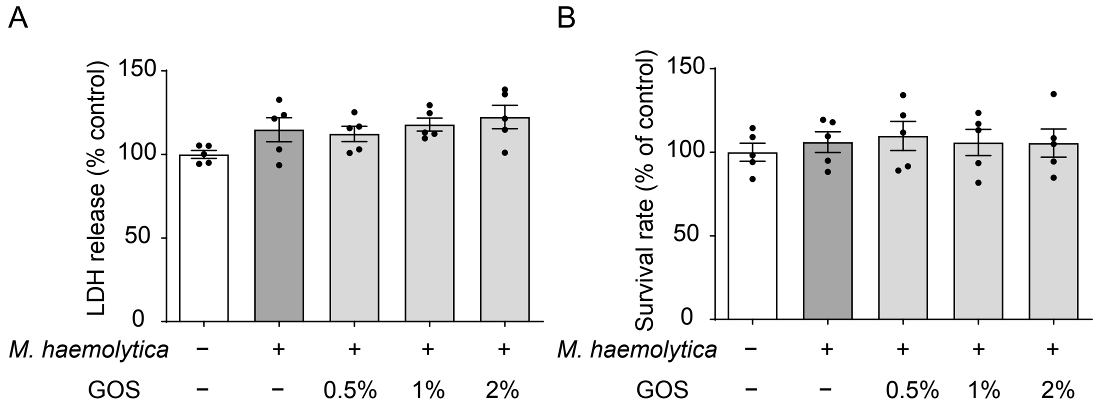

Figure S2. *M. haemolytica* with or without GOS does not affect the LDH release and survival rates in primary bronchial epithelial cells. PBECs

were incubated with *M. haemolytica* (1 x10^5 CFU/mL) for 24h with or without 24h pretreatment with GOS. (**A**) LDH release was assessed in the supernatants of PBECs. (**B**) Survival rates were determined by the percentage of MTT levels in PBECs. Data are presented as means ± SEM. All data shown are representative of at least five independent experiments (n=5 donor calves). GOS = galacto-oligosaccharides; LDH = lactate dehydrogenase; MTT = thiazolyl blue tetrazolium bromide; PBECs = primary bronchial epithelial cells.

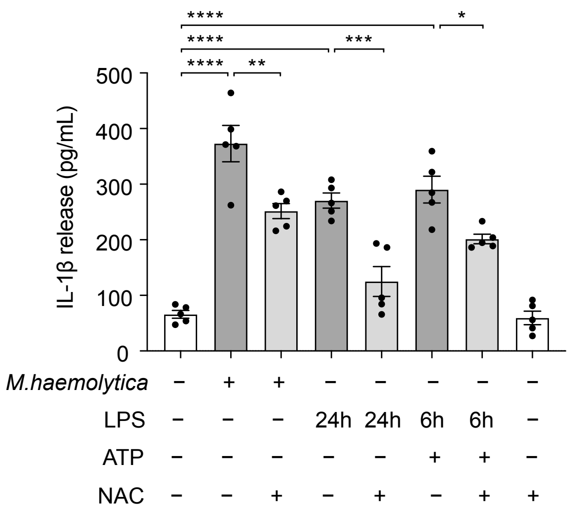

Figure S3. Inhibition of *M. haemolytica*/LPS/ATP-induced IL-1β release in primary bronchial epithelial cells by NAC. PBECs were incubated with *M. haemolytica* (1 x10^5 CFU/mL, 24h) or LPS (10 µg/mL, 24h) or LPS+ATP (10 µg/mL + 5 mM, 6h + 0.5h) with or without 6h pretreatment with 1mM NAC. The IL-1β release was determined by ELISA in the supernatants. **P*<0.05; ***P*<0.01; ****P*<0.001; *****P*<0.0001. Data are presented as means ± SEM. All data shown are representative of at least five independent experiments (n=5 donor calves). ATP = adenosine triphosphate; GOS = galacto-oligosaccharides; IL = interleukin; LPS = lipopolysaccharide; NAC = acetylcysteine; PBECs = primary bronchial epithelial cells.

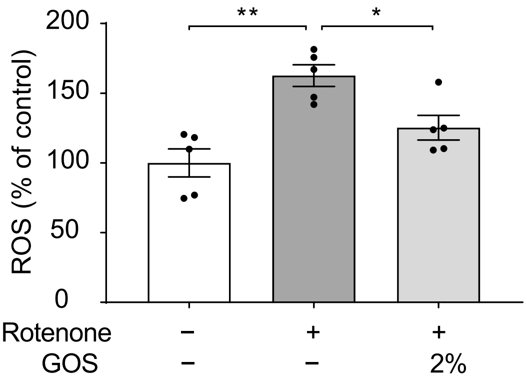

Figure S4. Inhibition of rotenone-induced mitochondrial ROS production in primary bronchial epithelial cells by GOS. PBECs were incubated with 10 μM rotenone for 6h with or without 24h GOS pretreatment. The production of mitochondrial ROS in PBECs was assessed and data were shown as a percentage of control. *P<0.05; **P<0.01. Data are presented as means ± SEM. All data shown are representative of at least five independent experiments (n=5 donor calves). GOS = galacto-oligosaccharides; ROS = reactive oxygen species; PBECs = primary bronchial epithelial cells.

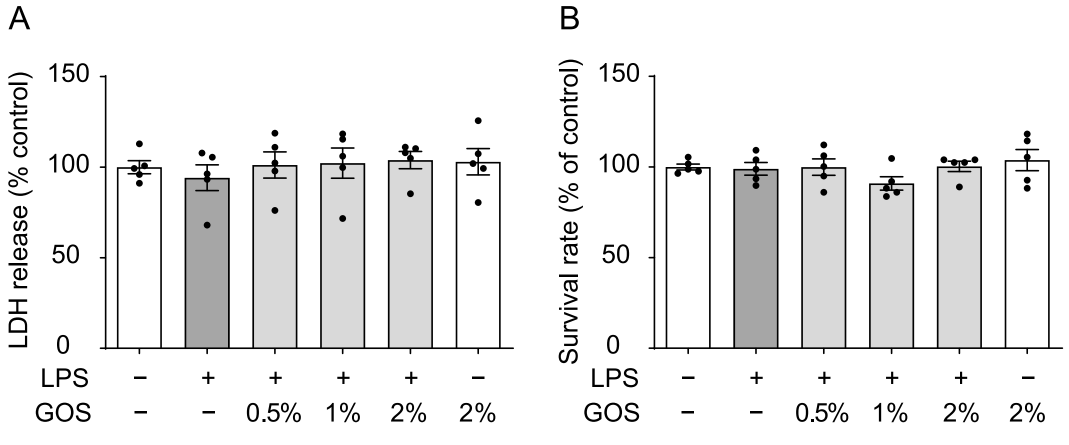

Figure S5. GOS with or without LPS do not affect the LDH release and survival rates in primary bronchial epithelial cells. PBECs were treated with 10 μg/mL LPS for 24h with or without 24h GOS pretreatment. (**A**) LDH release

was measured in the supernatants of PBECs. (**B**) Survival rates were determined by the percentage of MTT levels in PBECs. Data are presented as means ± SEM. All data shown are representative of at least five independent experiments (n=5 donor calves). GOS = galacto-oligosaccharides; LDH = lactate dehydrogenase; LPS = lipopolysaccharide; MTT = thiazolyl blue tetrazolium bromide; PBECs = primary bronchial epithelial cells.

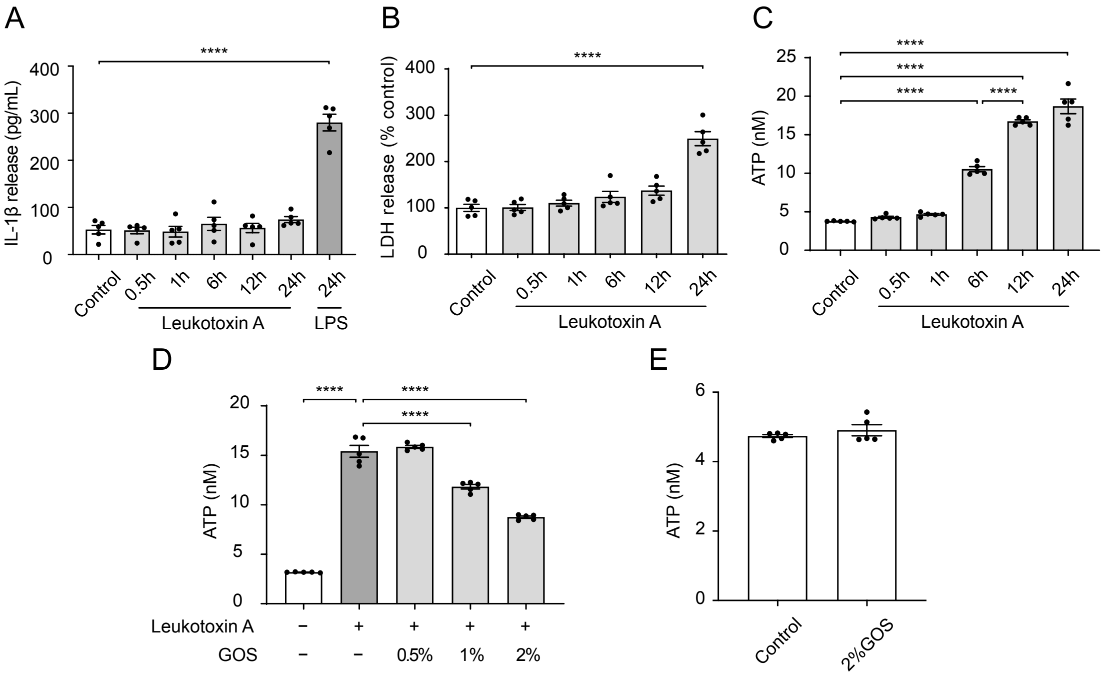

Figure S6. Inhibition of leukotoxin A-induced ATP production in primary bronchial epithelial cells by GOS. (**A**) PBECs were incubated with leukotoxin A for different time points (0.5, 1, 6, 12 and 24h) or LPS for 24h, and the IL-1β release was measured in the supernatants. (**B**-**C**) PBECs were incubated with or without leukotoxin A for different time points (0.5, 1, 6, 12 and 24h) and the LDH release and ATP production were examined. (**D**) PBECs were incubated with or without leukotoxin A for 12h with or without the 24h pretreatment with GOS and the ATP production was analyzed. (**E**) PBECs were treated with or without GOS for 48h, then ATP production in PBECs was assessed and data

were shown as an absolute amount. ****$P<0.0001$. Data are presented as means ± SEM. All data shown are representative of at least five independent experiments (n=5 donor calves). ATP = adenosine triphosphate; GOS = galacto-oligosaccharides; IL = interleukin; LPS = lipopolysaccharide; LDH = lactate dehydrogenase; PBECs = primary bronchial epithelial cells.

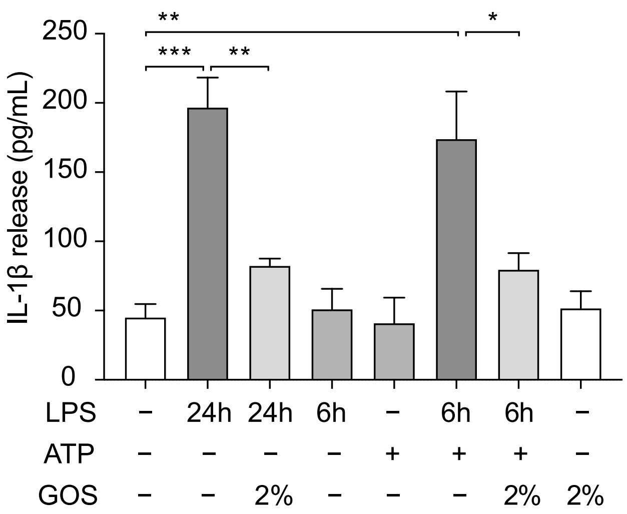

Figure S7. Inhibition of LPS- and LPS+ATP-induced IL-1β release in human bronchial epithelial cells by GOS. Human bronchial epithelial cells (16HBE) were incubated with LPS (10 µg/mL, 6h or 24h) or ATP (5 mM, 0.5h) or LPS+ATP (10 µg/mL + 5 mM, 6h + 0.5h) with or without 24h pretreatment with 2% GOS. The IL-1β release was determined by ELISA in the supernatants. *$P<0.05$; **$P<0.01$; ***$P<0.001$. Data are presented as means ± SEM. All data shown are representative of at least three independent experiments (n=3 cell generations). ATP = adenosine triphosphate; GOS = galacto-oligosaccharides; IL = interleukin; LPS = lipopolysaccharide.

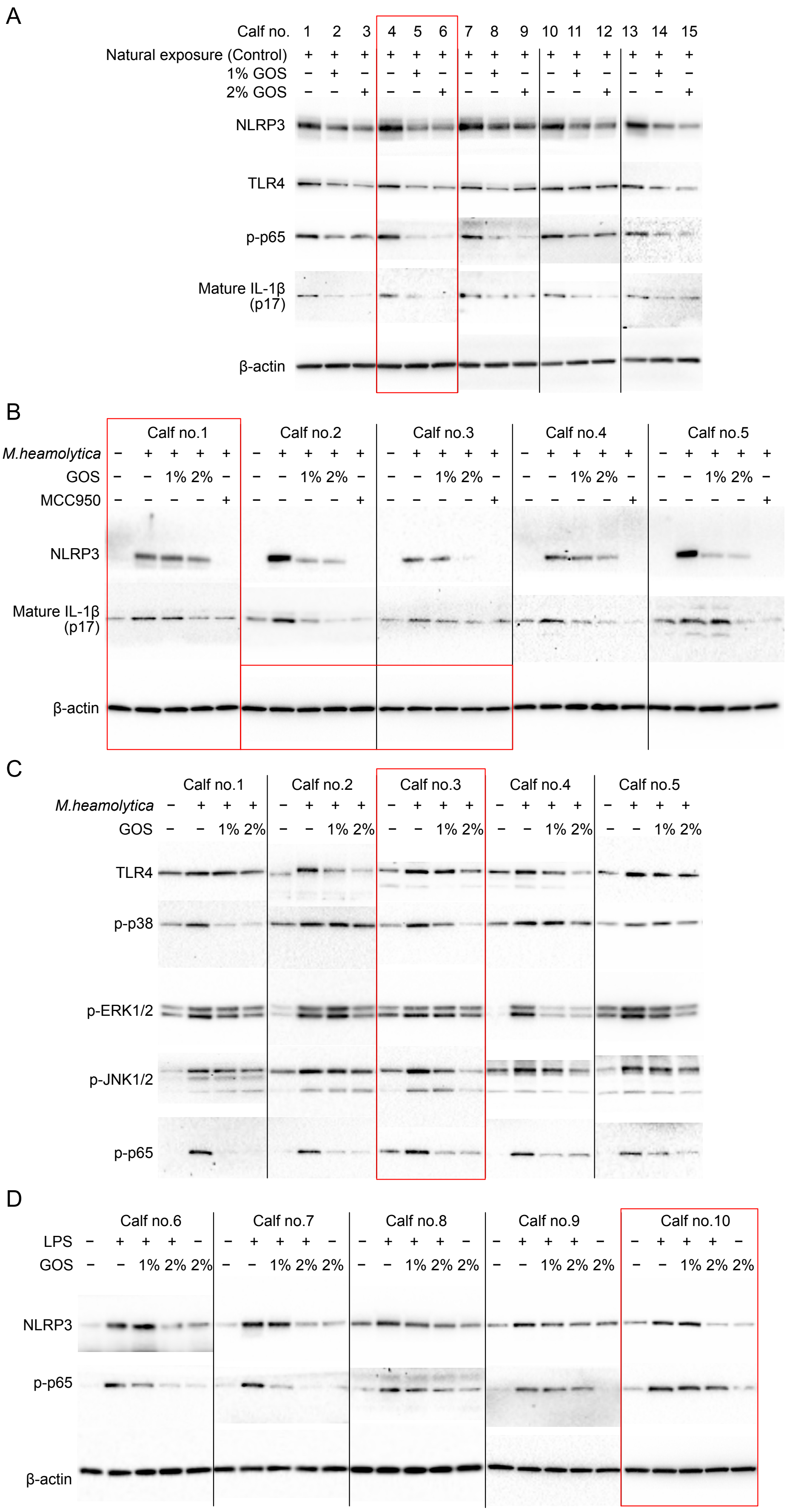

A
Calf no. 1 2 3 4 5 6 7 8 9 10 11 12 13 14 15
Natural exposure (Control)
1% GOS
2% GOS
NLRP3
TLR4
p-p65
Mature IL-1β (p17)
β-actin
B
Calf no.1
Calf no.2
Calf no.3
Calf no.4
Calf no.5
M.heamolytica
GOS
MCC950
1% 2%
NLRP3
Mature IL-1β (p17)
β-actin
C
Calf no.1
Calf no.2
Calf no.3
Calf no.4
Calf no.5
M.heamolytica
GOS
1% 2%
TLR4
p-p38
p-ERK1/2
p-JNK1/2
p-p65
D
Calf no.6
Calf no.7
Calf no.8
Calf no.9
Calf no.10
LPS
GOS
1% 2% 2%
NLRP3
p-p65
β-actin

Figure S8. Original blots of figures 3A, 5A, 5G, 5I, and 6E. Figure A represents the original blots of figure 3A (n=15, 5 calves/group). Figure B shows the original blots of figure 5A and G (n=5 donor calves). Figure C shows the original blots of figure 5I (n=5 donor calves). Figure D shows the original blots of figure 6E (n=5 donor calves). The original blots with the red boxes represent the images as shown in figure 3A, 5A, 5G, 5I, and 6E.

Supplemental Reference

1. Leruste, H., et al., *The relationship between clinical signs of respiratory system disorders and lung lesions at slaughter in veal calves.* Preventive Veterinary Medicine, 2012. **105**(1-2): p. 93-100.
2. McGuirk, S.M. and S.F. Peek, *Timely diagnosis of dairy calf respiratory disease using a standardized scoring system.* Anim Health Res Rev, 2014. **15**(2): p. 145-7.
